# Vegetative phenologies of lianas and trees in two Neotropical forests with contrasting rainfall regimes

**DOI:** 10.1111/nph.18150

**Published:** 2022-04-26

**Authors:** José A. Medina‐Vega, S. Joseph Wright, Frans Bongers, Stefan A. Schnitzer, Frank J. Sterck

**Affiliations:** ^1^ Forest Ecology and Forest Management Group Wageningen University and Research Centre Wageningen 6708 PB the Netherlands; ^2^ Smithsonian Tropical Research Institute Apartado Postal 0843‐03092 Balboa Ancón Panama; ^3^ Forest Global Earth Observatory Smithsonian Tropical Research Institute PO Box 37012 Washington DC 20013 USA; ^4^ Department of Biological Sciences Marquette University PO Box 1881 Milwaukee WI 53201 USA

**Keywords:** canopy, lianas, light, Panama, phenology, seasonality, trees, water potential

## Abstract

- Among tropical forests, lianas are predicted to have a growth advantage over trees during seasonal drought, with substantial implications for tree and forest dynamics. We tested the hypotheses that lianas maintain higher water status than trees during seasonal drought and that lianas maximize leaf cover to match high, dry‐season light conditions, while trees are more limited by moisture availability during the dry season.
- We monitored the seasonal dynamics of predawn and midday leaf water potentials and leaf phenology for branches of 16 liana and 16 tree species in the canopies of two lowland tropical forests with contrasting rainfall regimes in Panama.
- In a wet, weakly seasonal forest, lianas maintained higher water balance than trees and maximized their leaf cover during dry‐season conditions, when light availability was high, while trees experienced drought stress. In a drier, strongly seasonal forest, lianas and trees displayed similar dry season reductions in leaf cover following strong decreases in soil water availability.
- Greater soil moisture availability and a higher capacity to maintain water status allow lianas to maintain the turgor potentials that are critical for plant growth in a wet and weakly seasonal forest but not in a dry and strongly seasonal forest.

## Introduction

Lianas (woody vines) are an important plant lifeform in tropical forests, and are second only to trees in terms of biomass and contribution to leaf area (Gentry, 1992; Schnitzer & Bongers, 2002). Lianas use trees for support and access to high‐light positions in the forest canopy. Lianas compete intensely with trees for light and soil resources (Schnitzer, 2005; Toledo‐Aceves, 2015; Rodríguez‐Ronderos *et al*., 2016) and reduce tree regeneration (Schnitzer & Carson, 2010), growth (van der Heijden *et al*., 2015, 2019), reproduction (Wright *et al*., 2015; García León *et al*., 2018) and survival (Ingwell *et al*., 2010; Wright *et al*., 2015). Among tropical forests, liana abundance and diversity increase, both in absolute terms and relative to trees, with decreasing mean annual precipitation and increasing strength of seasonal drought (DeWalt *et al*., 2010; Parolari *et al*., 2020). Schnitzer (2005) advanced the seasonal growth advantage hypothesis (SGA) as a potential explanation for the pattern of increasing abundance and diversity of lianas with decreasing moisture availability. The SGA hypothesis posits that lianas maintain a more favorable water balance and thus grow more than co‐occurring trees during the drier season, when water availability is low (Schnitzer, 2005, 2018). Consequently, lianas are expected to accumulate additional annual growth in seasonal tropical forests, ultimately resulting in higher liana abundance than in wet, aseasonal forests, where lianas lack a seasonal growth advantage (Schnitzer, 2005, 2018). Indeed, lianas appear to grow more rapidly than trees during seasonal drought (Schnitzer & van der Heijden, 2019); however, the mechanisms responsible for the higher degree of growth observed in lianas than in trees during seasonal drought are not well understood.

In tropical forests, seasonal light availability is hypothesized to limit productivity and influence vegetative phenology (Van Schaik *et al*., 1993; Wright & Van Schaik, 1994). Above the canopy, light availability increases as cloud cover decrease from wet to dry season in most tropical forests (Windsor, 1990; Graham *et al*., 2003). Tropical tree species with access to dry‐season water supplies often regulate the production of leaves to match peaks of high, dry‐season irradiance (Wright & Van Schaik, 1994). Similarly, leaf production and forest productivity (i.e. photosynthesis) peak in the high‐light dry season in evergreen Amazônian and Asian monsoon forests (Elliott *et al*., 2006; Huete *et al*., 2006; Morton *et al*., 2014; Wu *et al*., 2016). Plants optimize photosynthetic carbon gain by producing new leaves and increasing total leaf area when irradiance is maximal (Field & Mooney, 1983; Kikuzawa, 1995; Kitajima *et al*., 1997; Doughty & Goulden, 2008). Correlations between leaf production, productivity, and irradiance suggest that light limits many tropical forest trees (Graham *et al*., 2003; Nemani *et al*., 2003). Nevertheless, not all trees and forests benefit from the abundant light availability during seasonal drought. In drier seasonal forests (< 2000 mm yr^−1^), tree species with different drought sensitivities show contrasting phenologies (Grubb, 1998; Eamus, 1999; Guan *et al*., 2015). Drought‐tolerant trees maintain hydraulic integrity via deep rooting and access to sufficient soil water and are predicted to produce leaves in the dry season (Van Schaik *et al*., 1993; Nepstad *et al*., 1994; da Rocha *et al*., 2004). Drought‐sensitive trees are predicted to produce leaves in the wet season because low water availability in the dry season induces strong water stress and limits plant function. The seasonal light availability hypothesis (SLA) predicts greater leaf production in the season of lowest cloud cover and highest irradiance among plants in forests with adequate water supplies (Van Schaik *et al*., 1993; Wright & Van Schaik, 1994).

The SGA and SLA hypotheses together suggest that lianas tend to have more access to dry‐season water supplies than trees, allowing lianas to maintain turgor potentials and regulate leaf production to match peaks of high, dry season irradiance. A more active leaf area during seasonal drought in lianas may optimize carbon gain (Field & Mooney, 1983, p. 198; Kikuzawa, 1995; Kitajima *et al*., 1997; Doughty & Goulden, 2008) and enable high growth rates (Borchert, 1999; Worbes, 1999; Schongart *et al*., 2002; O’Brien *et al*., 2008). The available evidence supports more favorable water status and higher physiological activity in lianas than in trees during seasonal drought. Lianas show higher leaf water potentials and photosynthetic rates than trees in the drier season in a seasonal tropical forest in China (Cai *et al*., 2009; Zhu & Cao, 2009; Chen *et al*., 2015) and in a common garden experiment in central Panama (Smith‐Martin *et al*., 2019) (Smith‐Martin *et al*., 2019). Lianas also allocate a higher proportion of their carbon to leaves (vs the stems) than trees, resulting in a relatively high photosynthetic tissue mass : plant mass ratio (Wyka *et al*., 2013; Medina‐Vega *et al*., 2021b), which, combined with high physiological activity during drought, may lead to increasingly high growth rates in dry periods. In central Panama, liana species tend to be evergreen and to produce new leaves during most of the year (Putz & Windsor, 1987), liana saplings grow faster in terms of height than tree saplings in the dry season (Schnitzer, 2005), and canopy lianas grow more rapidly in diameter than canopy trees during the dry season (Schnitzer & van der Heijden, 2019). These findings of these studies – that higher growth rates are observed in lianas than in trees during seasonal drought – support the fundamental premise of the SGA. However, the mechanisms that allow lianas to maintain hydraulic integrity and grow better than co‐occurring trees during dry periods remain unclear.

Previous studies suggest several possible mechanisms. Lianas may have deep (Holbrook & Putz, 1996; Restom & Nepstad, 2004; Andrade *et al*., 2005; Toledo‐Aceves & Swaine, 2008) and/or shallow lateral roots (De Deurwaerder *et al*., 2018; Smith‐Martin *et al*., 2019). Deep roots may enable lianas to access deep soil water (Schnitzer, 2005) and shallow lateral roots to efficiently capture dry‐season precipitation (De Deurwaerder *et al*., 2018), resulting in high hydraulic integrity and growth during seasonal drought (Schnitzer, 2005). High stem water storage (i.e. capacitance) resulting from abundant nonlignified parenchyma could also allow lianas to maintain hydraulic integrity during dry periods (Angyalossy *et al*., 2012; Isnard & Feild, 2015). Low resistance to hydraulic flow (i.e. high water transport capacity) resulting from their wide and long xylem elements (Ewers *et al*., 1991) may allow lianas to reduce water potential differences between soil and leaves, enabling high tissue water potentials and positive turgor potentials during drought. High water use efficiency resulting from strong stomatal control (Cai *et al*., 2009; Chen *et al*., 2015) may allow lianas to maximize gas exchange and growth while preventing excessive dehydration (see also Mumbanza *et al*., 2021). Additionally, a high accumulation of nonstructural carbohydrates for osmoregulation and hydraulic function (O’Brien *et al*., 2014; Martínez‐Vilalta *et al*., 2016) and reductions in leaf area (Lambers & Oliveira, 2019) could also explain the maintenance of high water status and turgor potentials in lianas during seasonal drought. Most of these possible mechanisms are mutually compatible and could reinforce one another.

Here, we explore predictions of the SGA and SLA hypotheses for the seasonal dynamics of leaf water status and leaf phenology of lianas and trees in the canopy of two lowland tropical forests on opposite sides of the Isthmus of Panama. One forest is relatively dry, strongly seasonal, and largely dry‐season deciduous. The second forest is wetter, weakly seasonal, and evergreen. We quantify the effects of seasonality on the hydraulic status and leaf phenology of eight liana and eight tree species in each forest (32 species in total). We tested three nonmutually exclusive hypotheses that address the fundamental premises of the SGA and SLA hypotheses. The first hypothesis is that predawn leaf water potentials (Ψ_pd_), which are indicative of access to soil water (Sala *et al*., 1981; Williams & Araujo, 2002; Santesteban *et al*., 2019), are higher in lianas than in trees during seasonal drought. The second hypothesis is that midday leaf water potentials (Ψ_md_), which would provide the positive turgor potential necessary for leaf development (see Boyer & Silk, 2004), are higher in lianas than in trees during seasonal drought. The third hypothesis is that the seasonal timing of leaf development differs between lianas and trees and that lianas tend to maximize leaf cover to match high, dry‐season light conditions, while trees tend to be more limited by moisture availability during the dry season. We test these hypotheses in each forest and discuss the potential mechanisms that might allow lianas to maintain hydraulic integrity and thus to grow better than co‐occurring trees during seasonal drought.

## Materials and Methods

### Study sites

**Fig. 1:**
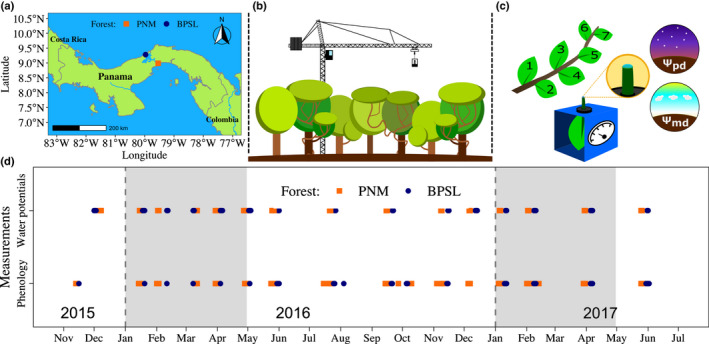
Location of the study sites and overview of the study design. (a) The location of the dry Parque Natural Metropolitano (PNM, orange square) and wet Bosque Protector San Lorenzo (BPSL, dark blue circle). (b) Graphical representation of the canopy cranes towering above the forest canopy, which were used at both study sites. (c) Graphical representation of a sample branch, with its leaves numbered, and a pressure chamber used to measure leaf water potential before dawn (predawn; Ψ_pd_) and during the hour following midday (midday; Ψ_md_) for both lianas and trees in both forests. (d) Plot showing the timing of vegetative phenology and leaf water potential data collection for both lianas and trees from the canopy cranes in PNM (orange squares) and BPSL (dark‐blue circles). Gray shading identifies the January‐to‐April dry season.

We collected data from two canopy cranes located in the Parque Natural Metropolitano (PNM, lat. 8°59′41.55″N, long. 79°32′35.22″W, 30 m above sea level (asl)) near Panama's Pacific coast and the Bosque Protector San Lorenzo (BPSL, lat. 9°16′51.71″N, long. 79°58′28.27″W, 130 m asl) near Panama’s Caribbean coast (Fig. 1a). Both cranes are equipped with a gondola suspended by cables from a trolley connected to a rotating jib (Fig. 1b). The 42‐m tall PNM crane had a 51 m jib and accessed 0.81 ha of forest. The 52‐m tall BPSL crane had a 54 m jib and accessed 0.91 ha of forest.

**Fig. 2:**
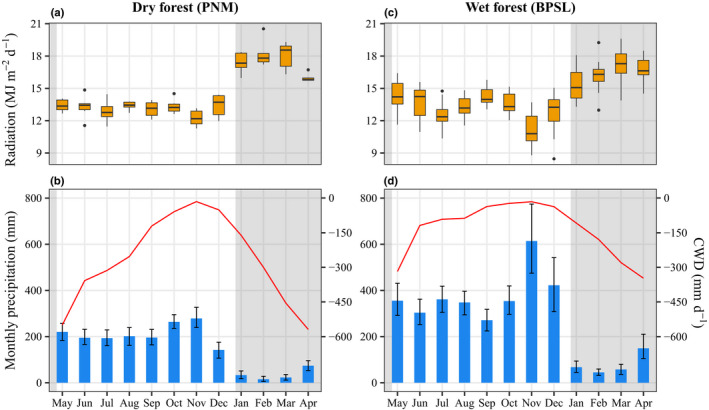
Environmental variables for the dry Parque Natural Metropolitano (PNM) and wet Bosque Protector San Lorenzo (BPSL) forests. Upper panels: Mean monthly solar radiation in the dry (a) and wet (c) forest. Box plots in (a, c) indicate (from bottom to top) the minimum (lower whiskers), lower quartile (Q1), median (horizontal lines), upper quartile (Q3), and maximum (upper whiskers) recorded monthly solar radiation. The points outside the range of the box plots represent outliers. Lower panels: Mean monthly precipitation (blue bars) and average minimum cumulative water deficits (CWD, red lines) for the dry (b) and wet (d) forests. Error bars in (b, d) indicate bootstrap ±SE. Gray shading identifies the January‐to‐April dry season. Horizontal axes indicate the month. Measurements were made at the cranes. Solar radiation is for 2012–2017 for the PNM and 2002, 2003, and 2008–2017 for the BPSL. Precipitation is for 1995–2017 for the PNM and 1997–2017 for the BPSL. Cumulative water deficit is for 2012–2017 for both forests. Cumulative water deficit is the cumulative difference between precipitation and potential evapotranspiration (PET). Data provided by the Physical Monitoring Program of the Smithsonian Tropical Research Institute.

The study sites experience contrasting rainfall regimes (Fig. 2). There is a drier season from mid–late December to mid–late April throughout Panama; however, the Caribbean sources most rain, and there is a strong Caribbean‐to‐Pacific rainfall gradient. At the BPSL and PNM sites, annual rainfall averages 3292 and 1864 mm, respectively, January‐through‐April rainfall averages 336 and 153 mm, and minimum cumulative dry‐season water deficits average −347 and −593 mm (Fig. 2). A stream at the base of the BPSL crane ceases to flow in some years, but streamside pools have retained water since the crane was installed in May 1997. The different dry‐season severities and soil conditions shape the different tree species compositions at both sites (Condit *et al*., 2013). Seasonality is much stronger at the PNM, where *c*. 50% of the canopy tree species are dry‐season deciduous (Wright, 2020). Seasonal changes in cloud cover cause seasonal changes in atmospheric transmissivity and solar radiation reaching both forests (Fig. 2). Mean temperatures are 25.4 and 26.1°C at the BPSL and PNM sites, respectively. The PNM site will hereafter be referred to as the ‘dry’ forest and the BPSL site as the ‘wet’ forest (Holdridge, 1967; Murphy & Lugo, 1986).

### Species selection and census protocol

We randomly selected eight liana and eight tree species at each site from species with two or more canopy individuals present. The 32 species included 22 families, with no species overlap between sites (Supporting Information Table S1). We selected two individuals of each species and four healthy branches of each individual. To minimize the variation associated with light levels, we selected branches in full sunlight and prevented other branches from overtopping them. The branches initially ranged from 30 to 70 cm in length. We numbered every leaf on each branch (Fig. 1c) with a permanent marker in November 2015 and returned 13 times through to May 2017 to record leaf water status and leaf births (Fig. 1d; Table S2). To record leaf births, we numbered new, fully expanded leaves at each census. We included leaves on all new branches that developed within the originally selected branches. If an entire branch died, we selected a replacement branch, so every individual had at least four branches throughout the study. Branch death occurred due to the movement of the gondolas and other biotic factors (i.e. sloths and monkeys feeding and/or hanging from the branches or wind‐thrown trees and lianas).

### Measurements of leaf water potential

We used predawn leaf water potential (Ψ_pd_) as an indicator of access to soil moisture (Sala *et al*., 1981; Williams & Araujo, 2002; Santesteban *et al*., 2019) and midday leaf water potential (Ψ_md_) as an indicator of leaf hydration status when evaporative demand is maximal (Williams & Araujo, 2002; Marechaux *et al*., 2018). We monitored Ψ_pd_ and Ψ_md_ during each leaf census (Fig. 1d) and made two additional measurements for all species in December 2015 and December 2016 (Table S2). We collected five leaves from the top of each census individual, avoiding census branches, and placed them in a sealable plastic bag (from which the air had been expelled) with wet paper towels to limit transpiration. We placed the bagged leaves inside a cooler with cooling gel packs to keep temperatures relatively low and further limit transpiration. We measured water potentials within 30 min of collection for three randomly selected leaves using a Scholander pressure chamber (Model 1000; PMS Instruments, Albany, OR, USA). We recorded Ψ_pd_ (MPa) before sunrise from 04:30 h to 5:30 h and Ψ_md_ (MPa) from 12:00 h to 13:00 h (Fig. 1c). When deciduous species lost their leaves, we collected and measured the tip of a branch following the same procedure.

### Environmental variables

At both cranes, precipitation (mm), solar radiation (MJ m^−2^ d^−1^), air temperature (°C), and relative humidity (%) were recorded by permanent weather stations via the Physical Monitoring Program of the Smithsonian Tropical Research Institute (STRI). An electronic tipping bucket (Model TB4; Campbell Scientific Inc., Logan, UT, USA) recorded precipitation at 15 min intervals. Pyranometers (Li‐Cor Model LI200X Silicon Pyranometer; Campbell Scientific Inc.) recorded global solar radiation every 10 s and logged mean, minimum and maximum values every 15 min. The tipping buckets and pyranometers were located on top of the canopy cranes. Temperature and relative humidity probes (Model CS215; Campbell Scientific Inc.) located 25 m above the forest floor recorded air temperature and relative humidity values on the same schedule. We calculated the vapor pressure deficit (VPD, kPa) from relative humidity and temperature as described by Jones (2013). We calculated cumulative water deficit (CWD) as CWD*_n_* = CWD*_n_*_−1_ + (P*_n_* − PET*_n_*), where *n, n* ^th^ day of the year; P, precipitation (mm); PET, potential evapotranspiration (mm); and positive CWD*_n_*_−1_ is reset to zero. CWD*_n_* potentially ranges from zero when P has been consistently larger than PET (i.e. soil saturation) to large negative values during intense dry periods. We calculated PET as described by Penman (1948) using the package evapotranspiration v.1.14 (Guo *et al*., 2016, 2019) in R v.4.0.3 (R Core Team, 2021) (Methods S1). We assumed soil saturation (i.e. CWD = 0) at the wettest time of the year (November; Fig. 2) and started the calculation of CWD.

### Assessment of the seasonal dynamics of leaf water potentials

We quantified the seasonal changes in Ψ_pd_ and Ψ_md_ for each site as a function of lifeform (liana vs tree) and CWD for each census using multilevel models with normally distributed errors. A square‐root transformation was used to normalize the absolute values of the response variables, Ψ_pd_ and Ψ_md_. We completed the transformation by multiplying the transformed values by –1 to retain the original direction of the response, with more negative values indicating more negative leaf water potentials. We standardized CWD to *Z*‐scores, dividing the difference between each observation and its mean value by its SD. Higher standardized (or scaled) values of CWD indicate a higher degree of soil saturation and lower values indicate drier conditions. To test hypotheses one and two, we included the interaction between lifeform and CWD in the models. The models included individuals nested within species as random intercepts to account for the nested structure of the design, which includes repeated censuses on individuals. We included CWD as a random slope to allow the community‐level coefficient (fixed effect) of the covariate to vary among individuals and species. In a preliminary analysis, we observed that the intercept varied consistently from census to census (Notes S1). We thus included the categorical variable ‘census’ as an additional random intercept to absorb variation in the intercept that was unexplained by species and individual identity (Methods S2).

### Assessment of the seasonal dynamics of leaf phenology

We quantified the changes in the proportion of leaf cover on branches of lianas and trees during a full annual leaf phenological cycle. This approach allowed us to isolate seasonal variation while controlling variation associated with branch size and allocation differences. For each species, a full annual leaf phenological cycle started with the first census with increasing leaf cover in year one and ended at the census preceding the first census with increasing leaf cover in year two. We then standardized the number of leaves present on each branch by the maximum observed number of leaves present for that same branch during the annual cycle. Branches included in the standardization were surveyed at least four times (i.e. for newly added branches), with a maximum of 10 surveys and a median and a mode of eight surveys. The resulting proportional leaf cover puts all branches on the same scale, with values ranging from 0 (fully deciduous) to 1 (maximum observed leaf cover for that branch).

The proportion of leaf cover takes values from the closed unit interval (0, 1), is possibly skewed, and has nonzero probability at zero and one (Fig. S1). To analyze this nonnormal zero and one inflated distribution, we used zero/one inflated beta regression (ZOIB) (Ospina & Ferrari, 2010; Liu & Eugenio, 2018). The ZOIB model takes data in the closed unit interval (0, 1) and has two components: the beta distribution on (0, 1) and the Bernoulli distribution for the binary 0 and 1 responses. The ZOIB model has four parameters, the mean (*μ*) and precision (*ν*) for the beta distribution (continuous response), and *α* and *γ* for the zero and one inflation (discrete responses), respectively. *μ* and *ν* determine the location, skew, and spread of the beta distribution in the (0, 1) interval, *α* is the probability that zero or one occurs (zero‐one‐inflation probability), and *γ* is the probability that one occurs rather than zero (conditional one‐inflation probability). An advantage of the ZOIB model is that covariate effects can be estimated for either or both of the continuous (*μ* and *ν*) and discrete responses (*α* and *γ*). In our analyses, we quantified covariate effects for both the continuous and discrete responses.

We quantified changes in standardized leaf cover as a function of lifeform, CWD for each census date, and mean solar radiation for the 20 d before each census (Srad). In a preliminary analysis, we tested the association of standardized leaf cover with mean solar radiation for the 10, 20, and 30 d before each census. The results were robust using the three time windows, but models using either 20 or 30 d showed an improved predictive quality. We thus used 20 d. To test hypothesis three, we included the interactions between CWD and lifeform and between Srad and lifeform. Lianas and trees differed in the number of axillary shoots, with trees having multiple branching events and a more complex branching structure than lianas (Medina‐Vega *et al*., 2021b). Multiple branching affects leaf production (Yaish *et al*., 2010). To control for different numbers of axillary shoots at each census, we added the covariate number of axillary shoots per branch (sb). To control for differences in water supply, we added the covariates Ψ_pd_ (MPa) and Ψ_md_ (MPa) as a proxy for access to soil moisture (Sala *et al*., 1981; Williams & Araujo, 2002; Santesteban *et al*., 2019) and leaf hydration status (Williams & Araujo, 2002; Marechaux *et al*., 2018), respectively. Precipitation and VPD were strongly collinear with CWD, Srad, Ψ_pd_, and Ψ_md_, with variance inflation factors > 3.0 (Zuur *et al*., 2010), and were therefore excluded. We did not consider air temperature because it was relatively constant across seasons and sites and because it was included in the estimation of PET, CWD, and VPD.

Following the analyses of the seasonal dynamics of leaf water potentials, we standardized the continuous covariates to *Z*‐scores. Models included branches nested within individuals and species as random intercepts to represent the nested structure of the design and account for dependence among repeated censuses of each branch. We also included random slopes at the species level for CWD and Srad. These random slopes allowed us to account for interspecific variability in the responses as a function of CWD and Srad. We evaluated alternative models, including the main effects of lifeform, CWD, Srad, Ψ_pd_, Ψ_md_, sb, and the interaction between CWD and lifeform and between Srad and lifeform (Methods S3).

### Prior specifications, model selection, evaluation, and inference

We fitted all models in ‘Stan’ (Carpenter *et al*., 2017), which fits models using the Hamiltonian Monte Carlo (HMC) algorithm, with its interface to R (v.4.0.3; R Core Team, 2021) via rstan (v.2.21.2; Stan Development Team, 2020) using the package brms (v.2.14.4; Bürkner, 2017, 2018). We opted for the Bayesian approach because, to our knowledge, it provides more practical tools for the building and assessment of the ZOIB model, via the R package brms, than the frequentist approach. See Notes S2, S3 for a description, justification, and sensitivity assessment of the prior probability distributions for the models that best fitted the seasonal dynamics of leaf water potential and leaf phenology, respectively.

For each analysis, we constructed candidate models by removing model terms that did not contribute to the quality of the models, and the best fit model was selected using leave‐one‐out cross‐validation. We estimated each model using four chains of 5000 iterations, each with a warm‐up fraction of ½. We monitored Markov Chain mixing properties and checked parameter convergence graphically via traceplots of the estimated coefficients (Notes S4, S5) and by checking the Rhat metric (Gelman *et al*., 2013). We inspected the goodness‐of‐fit of the best fit models via posterior predictive model checks (Gabry *et al*., 2019), where predictions from the best fit model were compared to the observed data (Notes S6, S7). This process allowed us to assess any obvious discrepancies between the final model and the observed data. The results are presented using the median, and the uncertainty in the estimates are summarized using the 89% credible intervals (CIs) computed using the highest density interval (HDI) of posterior distributions, which favors probable over central values, and is recommended for nonsymmetric posterior distributions (Kruschke, 2014; McElreath, 2020).

## Results

We tracked the development of 13 764 leaves during a full annual phenological cycle of branches representing 32 species, including 6861 leaves in the dry forest and 6903 leaves in the wet forest.

### The seasonal dynamics of leaf water potentials

**Fig. 3:**
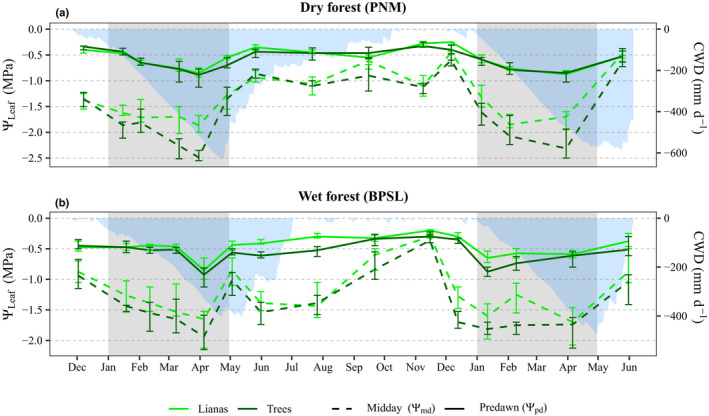
Median predawn (Ψ_pd_, solid lines) and median midday (Ψ_md_, dashed lines) leaf water potentials for lianas (light green) and trees (dark green) for the dry (PNM) (a) and wet (BPSL) (b) forest. Vertical bars show the 89% confidence interval of the lifeform‐level (i.e. lianas vs trees) median calculated from species‐level median values (i.e. raw data) using 1000 nonparametric bootstraps and the highest density interval method. Light blue areas in the background show cumulative water deficit (CWD) for reference. Gray shading indicates the January‐to‐April dry season. Vertical axes differ between panels and the original scales (–MPa) are used. Horizontal axes represent the time of the month at which the measurements were made.

For the dry forest, the seasonal dynamics of Ψ_pd_ were similar between lianas and trees, but the seasonal dynamics of Ψ_md_ differed between lifeforms (Fig. 3a). Ψ_pd_ increased consistently with increasing scaled values of CWD for both lifeforms (Table 1: *β_2_* in Model A; Fig. S2a). The hypothesis that lianas have higher Ψ_pd_ than trees (hypothesis one, see the Introduction section) is rejected for the dry forest. Ψ_md_ in trees consistently increased with increasing scaled values of CWD (Table 1: *β_3_* in Model C), while Ψ_md_ in lianas did not change with changes in CWD (Table 1: *β_2_* in Model C; Fig. S2b). The hypothesis that lianas have higher Ψ_md_ than trees (hypothesis two, see the Introduction section) is accepted for the dry forest. The best fit models for Ψ_pd_ and Ψ_md_ explained 69% (25% $R_{m}^{2}$, 69% $R_{c}^{2}$) and 79% (7% $R_{m}^{2}$, 79% $R_{c}^{2}$) of the variation in Ψ_pd_ and Ψ_md_ in the dry forest, respectively, with $R_{m}^{2}$and $R_{c}^{2}$representing the variation explained by fixed effects only and by fixed and random effects (Table S3) together, respectively.

**Table 1:** Summary of the fixed effects coefficients for the models that best fitted predawn (Ψ_pd_) and midday leaf water potentials (Ψ_md_) in the dry (PNM) and wet (BPSL) forests.

Fixed effects – coefficients	Dry forest – PNM			Wet forest – BPSL		
	Median	89CI lower	89CI upper	Median	89CI lower	89CI upper
Predawn leaf water potential Ψ_pd_	Model A			Model B		
*β_0_*– Intercept_sic_	**−0.745**	**−0.801**	**−0.686**	**−0.662**	**−0.719**	**−0.601**
*β_1_*– Lifeform tree_sic_	–	–	–	**−0.08**	**−0.133**	**−0.025**
*β_2_*– CWD_sic_	**0.087**	**0.043**	**0.136**	**0.088**	**0.037**	**0.136**
*β_3_*– Lifeform tree_sic_ : CWD_sic_	–	–	–	–	–	–
Midday leaf water potential Ψ_md_	Model C			Model D		
*β_0_*– Intercept_sic_	**−1.106**	**−1.242**	**−0.973**	**−1.065**	**−1.172**	**−0.962**
*β_1_*– Lifeform tree_sic_	−0.088	−0.187	0.007	**−0.089**	**−0.154**	**−0.025**
*β_2_*– CWD_sic_	0.034	−0.068	0.139	0.048	−0.048	0.147
*β_3_*– Lifeform tree_sic_ : CWD_sic_	**0.017**	**0.005**	**0.029**	–	–	–

A square‐root transformation was used to normalize the absolute values of the response variables Ψ_pd_ and Ψ_md_. We then multiplied the transformed values by –1 to recover the original direction of the response, with more negative values indicating more negative leaf water potentials. Median estimates that do not include zero within their credible intervals are in bold. Credible intervals were computed using the highest density interval (HDI) of posterior distributions, which is recommended for nonsymmetric (posterior) distributions (Kruschke, 2014). 89CI lower, lower 89% credible interval limit; 89CI upper, upper 89% credible interval limit; *β*_0…3_ estimated coefficients from Equation 1 in Supporting Information Methods S2; *c,* census; CWD_sic_, cumulative water deficit at the time of observation; *i,* individual; *s*, species. For each forest, we constructed candidate models by removing model terms (fixed and random effects) that did not contribute to the quality of the models, and the best fit model was selected using leave‐one‐out cross‐validation. Covariates that did not contribute to the model are indicated by a dash (‘–’). A coefficient that contains zero within the CIs indicates a negligible association between the covariate and the response variable at the community level (fixed effects; this table), but suggests an important variation in the response at the species level and/or individual level (see random effects in Table S3).

For the wet forest, lianas had higher Ψ_pd_ and Ψ_md_ than trees (Table 1: *β_1_* in Models B and D; Fig. S3). Hypotheses one and two are accepted for the wet forest. The best fit models for Ψ_pd_ and Ψ_md_ explained 58% (27% $R_{m}^{2}$, 58% $R_{c}^{2}$) and 65% (7% $R_{m}^{2}$, 65% $R_{c}^{2}$) of the variation in Ψ_pd_ and Ψ_md_ in the wet forest, respectively. See Figs S4–S7 for the species‐level estimates for Ψ_pd_ and Ψ_md_ in the dry and wet forests.

### The seasonal dynamics of leaf cover

The study branches were *c*. 30 m above ground level in the outermost canopy and thus supported the leaves with the longest path length from the root to the crown. These branches also experienced more direct sunlight, faster wind speeds, and greater evaporative demand than lower branches and leaves. Nonetheless, the observed phenological patterns in the sun‐lit branches were consistent with branches at lower levels within the same crown (J.A. Medina‐Vega, pers. obs.).

**Fig. 4:**
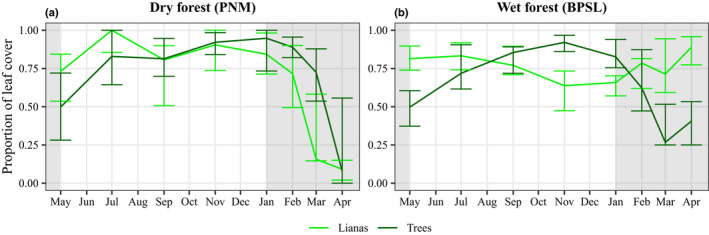
The (median) proportion of leaf cover in branches of lianas (light green) and trees (dark green) for the dry (PNM) (a) and wet (BPSL) (b) forests. Vertical bars show the 89% confidence interval of the lifeform‐level (i.e. lianas vs trees) median calculated from species‐level median values (i.e. raw data) using 1000 nonparametric bootstraps and the highest density interval method. Gray shading indicates the January‐to‐April dry season. Vertical axes represent the proportion of leaf cover. Horizontal axes represent the time of the month at which the measurements were made.

**Fig. 5:**
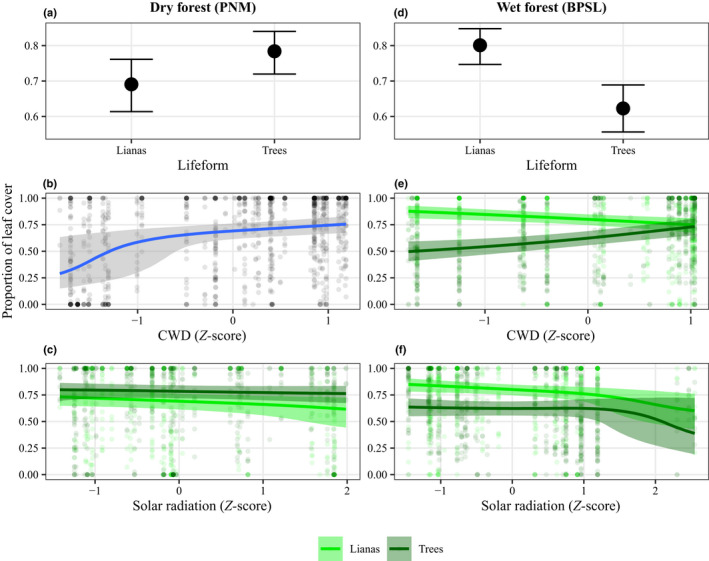
Conditional effects of lifeform (i.e. lianas vs trees) (a, d), cumulative water deficit (CWD) (b, e) and solar radiation (c, f) on the proportion of leaf cover of lianas and trees for the dry (PNM; left column) and wet (BPSL; right column) forests. The *y*‐axes show the *predicted* proportion of leaf cover. In (a) and (d), the *x*‐axes represent the levels of the categorical variable ‘lifeform’; in (b) and (e), they represent the (standardized) *Z*‐score of CWD (mm day^−1^); and in (c) and (f), they represent the (standardized) *Z*‐score of solar radiation (MJ m^−2^ d^−1^). The conditional effects for each predictor were calculated by setting the other (continuous) predictors to their mean values. The dots in (a) and (d) indicate the predicted median proportion of leaf cover for lianas and trees calculated from the posterior predictive distribution of the best‐fit model. The blue line in (b) indicates the predicted median proportion of leaf cover for both lianas and trees. The light green and dark green lines in (c), (e), and (f) indicate the predicted median proportion of leaf cover for lianas and trees, respectively. The error bars around the predicted median dot in (a) and (d), and the shadow(s) around the predicted median line(s) in (b), (c), (e) and (f) are represent the 89% credible intervals of the median estimate.

For the dry forest, the seasonal dynamics of leaf cover were similar for lianas and trees (Fig. 4a). Trees maintained a larger proportion of leaf cover than lianas (Fig. 5a). We observed a high degree of interspecific variation within lifeforms, broadly overlapping variation between lifeforms (error bars in Fig. 4a). Both lianas and trees were under strong hydraulic stress during seasonal drought due to their limited access to soil moisture (Figs 3a, 5b) and, presumably, high vapor pressure deficit. The hypothesis that lianas tend to maximize leaf cover to match high, dry‐season light conditions while trees tend to be more limited by moisture availability during the dry season (hypothesis three, see the Introduction section) is rejected for the dry forest (refer to Notes S8).

For the wet forest, the seasonal dynamics of leaf cover differed between lianas and trees (Figs 4b, 5e). Trees responded more strongly than lianas to changing light levels and water conditions (Fig. 5e,f). Lianas produced and maintained a higher proportion of leaf cover for longer, including during drier periods (Figs 4b, 5d,e). Hypothesis three is accepted for the wet forest.

For both forests, the covariate to control for multiple branching events (sb) made a consistent contribution to the quality of the models, and the best fit model for each forest explained a considerable amount of the variation in the proportion of leaf cover. An increase in the number of branching events was related to a consistent increase in the proportion of leaf cover (Table S4: *β_6_* in Model A and B for the dry forest and the wet forest, respectively) and to a higher probability of observing a fully covered branch for both lianas and trees in both forests (Table S4:*β_6_* in Model G and H for the dry forest and the wet forest, respectively). The fixed effects of the best fit model from the dry and wet forests explained 27% and 18% of the variation in the proportion of leaf cover (marginal *R* ^2^), and including random effects (Table S5), the explained variation increased to 52% and 47% (conditional *R* ^2^), respectively. (See Figs S8–S11 for the species‐level estimates of the proportion of leaf cover for lianas and trees in both the dry and wet forests.)

## Discussion

The SGA hypothesis and the SLA hypothesis are intimately related and together suggest that higher hydraulic integrity in lianas than in trees during dry periods allows lianas to maintain turgor potentials, which are critical for plant growth (Hsiao & Acevedo, 1974; Boyer & Silk, 2004), and to regulate leaf production to match peaks of high dry‐season irradiance. We found support for the SGA and SLA hypotheses in a wet, weakly seasonal, evergreen forest, but not in a dry, strongly seasonal, largely deciduous forest.

### Plant hydraulics support a liana seasonal growth advantage for a wet tropical forest

In the wet forest, lianas maintained higher water status than trees regardless of the season. Here, lianas had higher Ψ_pd_ than trees, which indicates greater access to soil water (Sala *et al*., 1981; Williams & Araujo, 2002; Santesteban *et al*., 2019) and may be particularly relevant in the dry season. The observed higher Ψ_pd_ in lianas than in trees in the wet forest is in line with other studies in a seasonal tropical forest in China (Zhu & Cao, 2009; Chen *et al*., 2015) and a common garden study in central Panama (Smith‐Martin *et al*., 2019) and with the hypothesis that lianas have a more efficient root architecture than trees (Schnitzer, 2005), by including deeper roots (Restom & Nepstad, 2004; Chen *et al*., 2015) and/or by having more shallow lateral roots (Johnson *et al*., 2013; De Deurwaerder *et al*., 2018). Lianas may thus benefit more from large volumes of potentially accessible soil water than trees (Smith‐Martin *et al*., 2019).

The higher Ψ_md_ in lianas in the wet forest indicates that they have lower hydric stress compared to trees (Williams & Araujo, 2002). Similar observations for lianas compared to trees in the dry season have been reported in a common garden study in central Panama (Smith‐Martin *et al*., 2019). For the wet forest, two independent studies, one using the same set of species (Medina‐Vega *et al*., 2021a) and another using a number of additional species (De Guzman *et al*., 2021), reported indistinguishable conductivities between terminal branches of lianas and trees. Moreover, it was reported that the branches of lianas and trees in this wet forest study site did not differ in terms of leaf area : sapwood area ratio (Medina‐Vega *et al*., 2021b). The lower hydric stress in lianas can thus not be attributed to a higher stem hydraulic conductivity, which in theory could allow lianas to benefit more from available soil water. Lianas and trees also did not differ in terms of hydraulic architecture, at least not in branches (see Medina‐Vega *et al*., 2021a), which are considered to be the bottlenecks in the hydraulic pathway of large plants (Anfodillo & Olson, 2021). These results contrast with those of multiple other studies, which showed that lianas had a higher xylem conductivity than trees (Ewers, 1985; Gartner *et al*., 1990; Ewers *et al*., 1991). It has been reported, in contrast to our findings, that canopy lianas have thinner stem diameters relative to the amount of foliage they supply than trees (Putz, 1983; Wyka *et al*., 2013). Such conflicting results may partially be explained by differences in sampling (see Rosell & Olson, 2014), since some of the studies reporting larger conductivities and higher leaf area : sapwood area ratios in lianas than in trees used more proximal stem segments (e.g. Ewers, 1985; Gartner *et al*., 1990; Ewers *et al*., 1991). While we cannot rule out the possibility that such differences in more proximal stem segments would also be observed for the lianas and trees of our study, the observed similarity in hydraulic conductivity and architecture between liana and tree branches (Medina‐Vega *et al*., 2021a,b) implies a remarkable convergence of hydraulic conductivity and architecture and cannot explain the difference in Ψ_md_ between lianas and trees in the wet forest.

The higher leaf water potentials in lianas suggest stronger stomatal control compared to trees, which allows lianas to reduce the variation in leaf water potentials by limiting transpiration (E) through reduced stomatal conductance (G_s_) (Tyree & Sperry, 1988; Cochard *et al*., 2002; Sperry *et al*., 2002). Since such stronger stomatal control may lead to a higher water use efficiency (the ratio of biomass accumulation to water lost) (Sinclair *et al*., 1984; Tardieu, 2013; Lawson & Blatt, 2014), this may allow the lianas under study here to maintain leaf productivity while preventing excessive dehydration under drier environmental conditions (Sinclair *et al*., 1984; Mumbanza *et al*., 2021). Such mechanisms may thus appear in the liana and tree communities under study here, as well as in liana communities in other forests (Andrade *et al*., 2005; Chen *et al*., 2015).

Another mechanism allowing lianas to achieve higher leaf productivity during dry periods would be increased osmoregulation, which has been observed in a liana–tree comparison in a tropical forest in French Guiana (Marechaux *et al*., 2017). Stronger osmoregulation would allow lianas to actively control their osmotic cell pressure by decreasing their osmotic potential (i.e. by accumulating osmotic solutes (glycinebetaine, sorbitol, and proline) (Lambers & Oliveira, 2019)) (Morgan, 1984; Hartmann & Trumbore, 2016), leading to the maintenance of turgor with decreasing water availability (Chaves *et al*., 2003; Lambers & Oliveira, 2019). In our study we did not measure proxies for osmoregulation, but in addition to a strong stomatal control, more active osmoregulation could also contribute to the maintenance of higher leaf water potentials, as observed for lianas compared to trees.

### Leaf phenology supports a liana seasonal growth advantage for a wet tropical forest

Lianas and trees in the wet forest showed contrasting patterns of leaf cover with seasonality. Lianas maintained their leaf cover during most of the year, while leaf cover in trees was constrained in the dry season (Fig. 4b). This result is consistent with that of another study conducted in central Panama, in which it was reported that liana species tend to be evergreen and produce new leaves during more of the year than tree species (Putz & Windsor, 1987). These results imply that higher leaf water potentials (Figs 3b; S3b,d) allowed the lianas to maintain and produce new leaves during seasonal drought, while the trees reduced their leaf cover during the same periods to avoid water stress (Figs 4b, 5e). The production of new leaf cover during seasonal drought may provide wet forest lianas with two advantages: they benefit from high dry‐season light levels (Fig. 2c) and they benefit from higher assimilation rates from younger than older leaves (Field & Mooney, 1983; Kikuzawa, 1995; Kitajima *et al*., 1997; Doughty & Goulden, 2008). A higher proportion of leaf cover (Fig. 4b) and higher water status in lianas than in trees during seasonal drought (Figs 3b; S3b,d) are consistent with both the SGA and SLA hypotheses.

### Hydraulics, vegetative phenology, and the seasonal growth advantage in a dry forest

In contrast to the wet forest, lianas and trees in the dry forest exhibited a similar response in Ψ_pd_ (Fig. 3a) and in changes in leaf cover with increasing drought intensity (Fig. 4a), but differed in terms of their Ψ_md_ responses (Fig. 3a). These results for the dry forest contrast with our expectation that lianas have greater access to soil water and a higher proportion of leaf cover than trees during seasonal drought.

The observation that lianas and trees in the dry forest experienced similar declines in Ψ_pd_ during seasonal drought contrasts with observations from the wet forest and with observations from a moist tropical forest in central Panama (Smith‐Martin *et al*., 2019) and a tropical dry forest in China (Chen *et al*., 2015). On‐site observations indicate that soils in the dry forest experience strong declines in water potentials (Santiago *et al*., 2004) and that the water table is deeper than 6 m in the dry season (B. Wolfe and M. Detto, pers. comm.). During seasonal drought, the low soil water supply may induce a strong convergence in Ψ_pd_ between lianas and trees in the dry forest.

Lianas in the dry forest experienced lower maximums of hydric stress (i.e. higher Ψ_md_) than trees in the dry season, implying greater stomatal control (Andrade *et al*., 2005; Chen *et al*., 2015). Additionally, increased osmoregulation (Marechaux *et al*., 2017) may contribute to lower hydric stress (Chaves *et al*., 2003; Lambers & Oliveira, 2019) in lianas than in trees, but this was not quantified in our study. However, the similar decrease in leaf cover between dry‐forest lianas and trees in the dry season (Fig. 4a) suggests that reducing leaf area is an important mechanism for maintaining hydraulic integrity during seasonal drought (Tyree *et al*., 1993). This finding is in agreement with those of another study at the same site, in which the cumulative proportion of the canopy surface of both lianas (three species overlap) and trees (six species overlap) was found to be lower in the dry season than in the wet season (Avalos & Mulkey, 1999). The shedding of leaves to avoid drought in tropical dry forests (i.e. mean annual rainfall < 2000 mm; Murphy & Lugo, 1986; Eamus, 1999; Guan *et al*., 2015) may therefore be a generic phenomenon for both trees and lianas at our dry forest study site (see Fig. S8), which contradicts the SGA and SLA hypotheses.

### The maintenance of hydraulic integrity in lianas

**Fig. 6:**
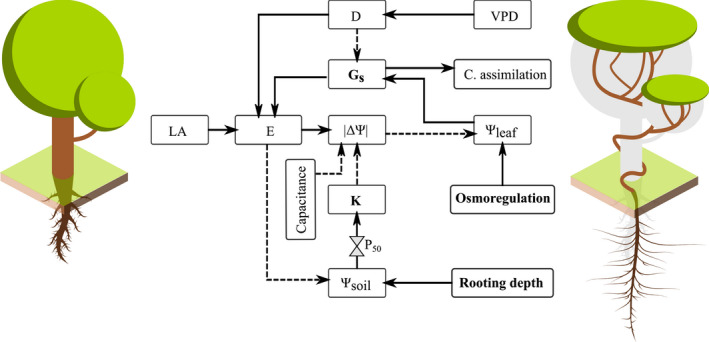
Schematic illustrating some of the variables and the relationships between plant hydraulics and carbon assimilation. Solid lines indicate positive relationships between variables, and dashed lines indicate negative relationships. Water flow and water status in the plant results from gradients in water potentials (ΔΨ) that originate from the interaction between soil water availability (Ψ_soil_) and transpiration (E). In dry periods, Ψ_soil_ drops and leaf‐to‐air vapor pressure difference (D) increases with increasing vapor pressure deficit (VPD). To maintain low differentials in water potentials (low |ΔΨ|) and physiological activity during seasonal drought, plants may connect strongly with water reserves (i.e. deeper and/or shallower lateral roots, conductance (K) and capacitance), plants may disconnect from the atmosphere with increasing D (i.e. reductions in stomatal conductance (G_s_) via stomatal control or reductions in leaf area (LA)), or plants may withstand and operate at high |ΔΨ| (i.e. greater resistance of the xylem to embolism (P_50_) and increased osmoregulation). Low |ΔΨ| leads to high leaf water potentials, turgor, and growth. Variables in bold are the suggested mechanisms that allow lianas to maintain water status, particularly during seasonal drought, and provide the turgor potentials needed to produce leaves and growth. See ‘The maintenance of hydraulic integrity in lianas’ in the Discussion section for further details.

Multiple mechanisms may allow lianas to maintain water status within tolerable limits and the turgor potentials critical for plant growth. The water potential of the plant is driven by gradients in water potential (ΔΨ) that result from the interaction between soil water potentials (Ψ_soil_), which govern water supply, and transpiration (E), which governs water loss (Dixon & Joly, 1895; Pickard, 1981) (Fig. 6). In wetter periods (i.e. high Ψ_soil_ and low VPD), plants maintain smaller gradients in water potentials (low |ΔΨ|) and higher leaf water potentials (Ψ_leaf_), which enable cell turgor and plant growth (Boyer & Silk, 2004; Muller *et al*., 2011), than in drier periods. In drier periods, Ψ_soil_ drops and leaf‐to‐air vapor pressure differences (D) increase with increasing VPD. High D and low Ψ_soil_ lead to high |ΔΨ| through increased E and reduced conductance (K). High |ΔΨ| leads to lower Ψ_leaf_, reduced cell turgidity, and reduced growth. It has been proposed that lianas may connect strongly to soil water via deep (Restom & Nepstad, 2004) and/or shallow lateral roots (Johnson *et al*., 2013; De Deurwaerder *et al*., 2018), and greater K may allow lianas to maintain lower |ΔΨ| by moving available soil water from their roots to their leaves more rapidly than trees (Ewers, 1985; Gartner *et al*., 1990; Ewers *et al*., 1991). With increasing drought, greater stomatal control (Andrade *et al*., 2005; Chen *et al*., 2015) may allow lianas to reduce variation in ΔΨ by limiting E through reduced stomatal conductance (G_s_) (Tyree & Sperry, 1988; Cochard *et al*., 2002; Sperry *et al*., 2002) and increased osmoregulation (Marechaux *et al*., 2017) may allow lianas to maintain turgor potentials and operate at high |ΔΨ| (Chaves *et al*., 2003; Lambers & Oliveira, 2019). These mechanisms are mutually compatible and could reinforce one another.

We discarded mechanisms such as capacitance, which could provide lianas with additional water availability (Ewers & Fisher, 1991; Spicer & Groover, 2010; Isnard & Feild, 2015), and embolism resistance (P_50_), which could allow lianas to operate under high evaporative demand, because their use is not supported by the literature. Previous studies from our study sites that included individuals from the same set of species as our study reported that the branches of lianas do not have higher capacitance (De Guzman *et al*., 2017, 2021) or higher embolism resistance than the branches of trees (De Guzman *et al*., 2021; Medina‐Vega *et al*., 2021a). Results from these studies suggest that lianas rely primarily on soil water availability and conductive capability (Chen *et al*., 2016; De Guzman *et al*., 2017), and in the absence of soil water, liana stems may be highly vulnerable to drought‐induced cavitation (P_50_) (Hacke *et al*., 2006; Gutierrez *et al*., 2009; van der Sande *et al*., 2013).

Some of the discussed mechanisms (i.e. G_s_, K, and osmoregulation (Fig. 6)) that may allow lianas to maintain hydraulic integrity during seasonal drought are not directly tested in this study. Neither does this study directly test the link between high hydraulic integrity during seasonal drought, additional annual growth, and a high or increasing abundance of lianas in seasonally dry tropical forests. Our study does imply, however, that better access to soil water – as implied by the SGA and SLA hypotheses – is not a sufficient explanation for the higher numbers of lianas in drier forests and that the mechanisms that maintain a small |ΔΨ| should be considered from a whole‐plant perspective (Fig. 6). We therefore call for studies that combine multiple physiological observations (see Fig. 6) and the seasonality of those observations (e.g. accumulation of osmotically active compounds), with detailed and more direct measurements of growth (e.g. diameter, height (length), biomass) and for longer time periods. These studies may provide a better picture of the mechanisms that allow lianas to grow more than trees during seasonal drought and explain the higher liana abundances in seasonal tropical forests (Schnitzer, 2005, 2018).

Our study included two forests with no species overlap between sites. Under the Panama rainfall gradient, dry‐season severity and soil P are the main drivers of tree species distribution (Condit *et al*., 2013), with *c*. 50% of canopy tree species in the dry forest being dry‐season deciduous (Wright, 2020). Resource availability and life history may also shape the spatial distribution of liana species under this gradient (see Medina‐Vega *et al*., 2021a,b), and these possibilities require further investigation. Given that the variations in cloud cover (i.e. light), precipitation (Fig. 2) and soil nutrients (Woodring *et al*., 1980; Santiago *et al*., 2005) under this gradient are consistent with broader geographical gradients across the tropics, our findings may apply to other dry and wet tropical forests. Remarkably, we found support for the SGA and SLA hypotheses in a wet, weakly seasonal, evergreen forest, but not in a dry, strongly seasonal, largely deciduous forest.

## Author contributions

JAM‐V, SJW, FB, SAS and FJS conceived the ideas and designed the methodology. JAM‐V collected and analyzed the data. JAM‐V and SJW interpreted the data. JAM‐V led the writing of the manuscript, with comments from SJW, FB, SAS and FJS. All authors contributed critically to the drafts and gave final approval for publication.

## Supporting information


**Fig. S1** Frequency distribution for the proportion of leaf cover.
**Fig. S2** Conditional effects for the models that best fitted leaf water potentials in the dry forest.
**Fig. S3** Conditional effects for the models that best fitted leaf water potentials in the wet forest.
**Fig. S4** Species‐level predictions for predawn leaf water potentials – dry forest.
**Fig. S5** Species‐level predictions for midday leaf water potentials – dry forest.
**Fig. S6** Species‐level predictions for predawn leaf water potentials – wet forest.
**Fig. S7** Species‐level predictions for midday leaf water potentials – wet forest.
**Fig. S8** Species‐level predictions – proportion of leaf cover as a function of cumulative water deficit in the dry forest.
**Fig. S9** Species‐level predictions – proportion of leaf cover as a function of CWD in the wet forest.
**Fig. S10** Species‐level predictions – proportion of leaf cover as a function of solar radiation in the dry forest.
**Fig. S11** Species‐level predictions – proportion of leaf cover as a function of solar radiation in the wet forest.
**Methods S1** Calculation of potential evapotranspiration.
**Methods S1** Regression equation – leaf water potential models.
**Methods S1** Regression equation – proportion of leaf cover models.
**Note S1** Justification for the inclusion of census as a random intercept in the models of leaf water potentials.
**Note S2** Prior justification – leaf water potential models.
**Note S3** Prior justification – proportion of leaf cover models.
**Note S4** Traceplots – leaf water potential models.
**Note S5** Traceplots – proportion of leaf cover models.
**Note S6** Posterior predictive checks – leaf water potential models.
**Note S7** Posterior predictive checks – proportion of leaf cover models.
**Note S8** Supplementary results – proportion of leaf cover models.
**Table S1** Study species.
**Table S2** Census dates.
**Table S3** Random effects – leaf water potential models.
**Table S4** Fixed effects – proportion of leaf cover models.
**Table S5** Random effects – proportion of leaf cover models.
Please note: Wiley Blackwell are not responsible for the content or functionality of any Supporting Information supplied by the authors. Any queries (other than missing material) should be directed to the *New Phytologist* Central Office.

## Data Availability

Data and code supporting the results are available from Zenodo: https://doi.org/10.5281/zenodo.6403252 (Medina‐Vega *et al*., 2022).
