# Supplementary material for: Vegetative phenologies of lianas and trees in two Neotropical forests with contrasting rainfall regimes
Source: New Phytol. 2022 Apr 26;235(2):457–71. doi: 10.1111/nph.18150 (PMC9325559; doi:10.1111/nph.18150)
Supplement: Supplementary file 1 — Fig. S1 Frequency distribution for the proportion of leaf cover. Fig. S2 Conditional effects for the models that best fitted leaf water potentials in the dry forest. Fig. S3 Conditional effects for the models that best fitted leaf water potentials in the wet forest. Fig. S4 Species‐level predictions for predawn leaf water potentials – dry forest. Fig. S5 Species‐level predictions for midday leaf water potentials – dry forest. Fig. S6 Species‐level predictions for predawn leaf water potentials – wet forest. Fig. S7 Species‐level predictions for midday leaf water potentials – wet forest. Fig. S8 Species‐level predictions – proportion of leaf cover as a function of cumulative water deficit in the dry forest. Fig. S9 Species‐level predictions – proportion of leaf cover as a function of CWD in the wet forest. Fig. S10 Species‐level predictions – proportion of leaf cover as a function of solar radiation in the dry forest. Fig. S11 Species‐level predictions – proportion of leaf cover as a function of solar radiation in the wet forest. Methods S1 Calculation of potential evapotranspiration. Methods S1 Regression equation – leaf water potential models. Methods S1 Regression equation – proportion of leaf cover models. Note S1 Justification for the inclusion of census as a random intercept in the models of leaf water potentials. Note S2 Prior justification – leaf water potential models. Note S3 Prior justification – proportion of leaf cover models. Note S4 Traceplots – leaf water potential models. Note S5 Traceplots – proportion of leaf cover models. Note S6 Posterior predictive checks – leaf water potential models. Note S7 Posterior predictive checks – proportion of leaf cover models. Note S8 Supplementary results – proportion of leaf cover models. Table S1 Study species. Table S2 Census dates. Table S3 Random effects – leaf water potential models. Table S4 Fixed effects – proportion of leaf cover models. Table S5 Random effects – proportion of leaf cover models. Please note: [file NPH-235-457-s001.pdf]

***New Phytologist* Supporting Information**

Article title: **Vegetative phenologies of lianas and trees in two Neotropical forests with contrasting rainfall regimes**.

Authors: José A. Medina-Vega, S. Joseph Wright, Frans Bongers, Stefan A. Schnitzer and Frank J. Sterck.

Article acceptance date: 27 March 2022.

The following Supporting Information is available for this article:

Table S1: Study species.

Table S2: Census dates.

Table S3: Random effects - leaf water potentials models.

Table S4: Fixed effects - proportion of leaf cover models.

Table S5: Random effects - proportion of leaf cover models.

Methods S1: Calculation of potential evapotranspiration (PET).

Methods S2: Regression equation - leaf water potential models.

Methods S3: Regression equation - proportion of leaf cover models.

Notes S1: Justification for the inclusion of census as random intercept in the models of leaf water potentials.

Notes S2: Prior justification - leaf water potential models.

Notes S3: Prior justification - proportion of leaf cover models.

Notes S4: Traceplots - leaf water potential models.

Notes S5: Traceplots - proportion of leaf cover models.

Notes S6: Posterior predictive checks - leaf water potential models.

Notes S7: Posterior predictive checks - proportion of leaf cover models.

Notes S8: Supplementary results - proportion of leaf cover models.

Fig. S1: Frequency distribution for the proportion of leaf cover.

Fig. S2: Conditional effects for the models that best-fitted leaf water potentials in the dry forest.

Fig. S3: Conditional effects for the models that best-fitted leaf water potentials in the wet forest.

Fig. S4: Species-level predictions for predawn leaf water potentials - dry forest.

Fig. S5: Species-level predictions for midday leaf water potentials - dry forest.

Fig. S6: Species-level predictions for predawn leaf water potentials - wet forest.

Fig. S7: Species-level predictions for midday leaf water potentials - wet forest.

Fig. S8: Species-level predictions - proportion of leaf cover as a function of CWD in the dry forest.

Fig. S9: Species-level predictions - proportion of leaf cover as a function of CWD in the wet forest.

Fig. S10: Species-level predictions - proportion of leaf cover as a function of solar radiation in the dry forest.

Fig. S11: Species-level predictions - proportion of leaf cover as a function of solar radiation in the wet forest.

Table S1: Study species classified by lifeform (lianas and trees) and the number of leaf births recorded over a period of $\sim$18 months for each species (added value for the set of monitored branches per species) in the dry (Parque Natural Metropolitano, PNM) and wet (Bosque Protector San Lorenzo, BPSL) forest.

Site	Growth form	Family	Species	Leaf births
**Dry forest (PNM)**	**Lianas**	Bignoniaceae	*Amphilophium crucigerum* (L.) L.G. Lohmann	220
		Bignoniaceae	*Callichlamys latifolia* (Rich.) K. Schum.	66
		Bignoniaceae	*Stizophyllum riparium* (Kunth) Sandwith	388
		Convolvulaceae	*Bonamia trichantha* Hallier f.	1069
		Malpighiaceae	*Stigmaphyllon hypargyreum* Triana & Planch.	1444
		Petiveriaceae	*Trichostigma octandrum* (L.) H.Walter	1110
		Sapindaceae	*Serjania mexicana* (L.) Willd.	161
		Vitaceae	*Vitis tiliifolia* Humb. & Bonpl. ex Schult.	621
	**Trees**	Anacardiaceae	*Anacardium excelsum* (Bertero & Balb. ex Kunth) Skeels	2822
		Anacardiaceae	*Astronium graveolens* Jacq.	888
		Annonaceae	*Annona spraguei* Saff.	776
		Boraginaceae	*Cordia alliodora* (Ruiz & Pav.) Oken	569
		Lauraceae	*Cinnamomum triplinerve* (Ruiz & Pav.) Kosterm.	360
		Malvaceae	*Guazuma ulmifolia* Lam.	2079
		Malvaceae	*Luehea seemannii* Triana & Planch	645
		Rubiaceae	*Pittoniotis trichantha* Griseb.	2027
**Wet forest (BPSL)**	**Lianas**	Bignoniaceae	*Pleonotoma variabilis* (Jacq.) Miers	167
		Celastraceae	*Salacia multiflora* (Lam.) DC.	49
		Celastraceae	*Tontelea passiflora* (Vell.) Lombardi	113
		Convolvulaceae	*Maripa panamensis* Hemsl.	390
		Dilleniaceae	*Doliocarpus multiflorus* Standl.	191
		Euphorbiaceae	*Omphalea diandra* L.	259
		Olacaceae	*Heisteria scandens* Ducke	616
		Polygonaceae	*Coccoloba excelsa* Benth.	83
	**Trees**	Annonaceae	*Guatteria dumetorum* R.E.Fr.	2827
		Combretaceae	*Terminalia amazonia* (J.F. Gmel.) Exell	2186
		Fabaceae	*Tachigali versicolor* Standl. & L.O.Williams	241
		Malvaceae	*Apeiba aspera* Aubl.	1037
		Melastomataceae	*Miconia minutiflora* (Bonpl.) DC.	1420
		Myristicaceae	*Virola multiflora* (Standl.) A.C. Sm.	1672
		Rubiaceae	*Tocoyena pittieri* (Standl.) Standl.	96
		Vochysiaceae	*Vochysia ferruginea* Mart.	899

Table S2: The number and start date of each census period in the dry (Parque Natural Metropolitano, PNM) and wet (Bosque Protector San Lorenzo, BPSL) forest. Census marked with an asterisks (*) are the two extra measurements of leaf water potentials.

Census	Dry forest (PNM)	Wet forest (BPSL)
1	November 11th, 2015	November 15th, 2015
*	December 7th, 2015	December 1st, 2015
2	January 14th, 2016	January 20th, 2016
3	February 1st, 2016	February 11th, 2016
4	March 8th, 2016	March 10th, 2016
5	March 30th, 2016	April 4th, 2016
6	April 28th, 2016	May 3rd, 2016
7	May 24th, 2016	May 30th, 2016
8	July 15th, 2016	July 25th, 2016
9	September 14th, 2016	September 20th, 2016
10	November 4th, 2016	November 14th, 2016
*	December 6th, 2016	December 12th, 2016
11	January 4th, 2017	January 10th, 2017
12	February 1st, 2017	February 8th, 2017
13	March 29th, 2017	April 5th, 2017
14	May 24th, 2017	May 30th, 2017

Table S3. Summary of the random effects for the models that best-fitted predawn (ψ_{pd}) and midday (ψ_{md}) leaf water potentials.

Summary of the random effects for the models that best-fitted predawn leaf water potentials (ψ_{pd}) and midday leaf water potential (ψ_{md}) in the dry (PNM) and wet (BPSL) forest.

	Dry forest - PNM			Wet forest - BPSL		
**Random effects**	σ	**89CI lower**	**89CI upper**	σ	**89CI lower**	**89CI upper**
**Predawn leaf water potential (ψ_{pd})**	**Model A**			**Model B**		
S_s	0.074	0.051	0.102	0.063	0.041	0.089
$\gamma_s - CWD$	0.022	0.006	0.036	0.015	0.000	0.027
$I_{i(s)}$	0.017	0.007	0.027	0.028	0.016	0.040
$\lambda_{i(s)} - CWD$	0.016	0.006	0.028	0.023	0.012	0.034
C_c	0.106	0.072	0.144	0.103	0.069	0.145
σ_{sic}	0.090	0.090	0.100	0.110	0.110	0.120
**Midday leaf water potential (ψ_{md})**	**Model C**			**Model D**		
S_s	0.110	0.072	0.155	0.080	0.050	0.112
$\gamma_s - CWD$	-	-	-	0.034	0.018	0.051
$I_{i(s)}$	0.049	0.033	0.070	0.036	0.020	0.053
$\lambda_{i(s)} - CWD$	-	-	-	0.012	0.000	0.027
C_c	0.263	0.185	0.362	0.217	0.148	0.296
σ_{sic}	0.130	0.130	0.140	0.170	0.160	0.170

A square-root transformation was used to normalize the absolute value of the response variables, predawn (ψ_{pd}) and midday leaf water potential (ψ_{md}). We then multiplied the transformed values by negative one to get the original direction of the response; more negative values indicating more negative leaf water potentials. σ is the median estimate of the (random effects) standard deviation calculated from the posterior distribution. '89CI lower' and '89CI upper' are the lower and upper 89% credible interval limits, respectively; computed using the highest density interval (HDI) of posterior distributions, which is recommended for non-symmetric (posterior) distributions (Kruschke, 2014). S_s is the random intercept for species. γ_s is the random slope of CWD at the species level. $I_{i(s)}$ is the random intercept for individuals nested within species. $\lambda_{i(s)}$ is random slope of CWD at the individual level. C_c is the random intercept for census. σ_{sic} is the residual standard deviation. Fixed effects are in Table 1 in the main text.

Table S4. Summary of the fixed effects for the models that best-fitted the proportion of leaf cover.

Summary of the fixed effects for the models that best-fitted the proportion of leaf cover on branches of lianas and trees in the dry (models A C, E and G, PNM) and wet (models B, D, F and H, BPSL) forest. Median estimates that do not include zero within their CIs are in bold.

	Dry forest - PNM			Wet forest - BPSL		
**Parameter**	**Median**	**89CI lower**	**89CI upper**	**Median**	**89CI lower**	**89CI upper**
**Mean - μ**	**Model A**			**Model B**		
β_0 - Intercept$_{sib}$	0.097	-0.298	0.459	**0.972**	**0.669**	**1.234**
β_1 - Lifeform Tree$_{sib}$	**0.61**	**0.131**	**1.197**	**-0.77**	**-1.16**	**-0.373**
β_2 - CWD$_{sib}$	**0.409**	**0.206**	**0.616**	**-0.206**	**-0.377**	**-0.045**
β_3 - Solar radiation$_{sib}$	0.043	-0.096	0.181	-0.131	-0.292	0.035
β_4 - Predawn leaf water potential$_{sib}$	-	-	-	-	-	-
β_5 - Midday leaf water potential$_{sib}$	-	-	-	-	-	-
β_6 - Nshoots$_{sib}$	**0.59**	**0.446**	**0.73**	**0.693**	**0.589**	**0.79**
β_7 - CWD : Lifeform.Tree$_{sib}$	-	-	-	**0.374**	**0.15**	**0.596**
β_8 - Lifeform.Tree : Solar radiation$_{sib}$	-	-	-	-	-	-
**Precision - ν**	**Model C**			**Model D**		
β_0 - Intercept$_{sib}$	**1.854**	**1.619**	**2.095**	**2.368**	**2.097**	**2.618**
β_1 - Lifeform Tree $_{sib}$	-	-	-	-	-	-
β_2 - CWD$_{sib}$	-0.112	-0.37	0.125	**-0.777**	**-1.16**	**-0.365**
β_3 - Solar radiation$_{sib}$	-0.071	-0.278	0.149	**-0.535**	**-0.792**	**-0.262**
β_4 - Predawn leaf water potential$_{sib}$	-	-	-	-	-	-
β_5 - Midday leaf water potential$_{sib}$	**-0.206**	**-0.393**	**-0.015**	**-0.205**	**-0.332**	**-0.074**
β_6 - Nshoots$_{sib}$	-	-	-	-	-	-
β_7 - CWD : Lifeform.Tree $_{sib}$	-	-	-	-	-	-
β_8 - Lifeform.Tree : Solar radiation$_{sib}$	-	-	-	-	-	-
**Zero-one inflation probability - α**	**Model E**			**Model F**		
β_0 - Intercept$_{sib}$	**-0.602**	**-0.973**	**-0.256**	**-1.007**	**-1.558**	**-0.442**
β_1 - Lifeform Tree $_{sib}$	-	-	-	-0.65	-1.42	0.15
β_2 - CWD$_{sib}$	-0.171	-0.387	0.063	-0.34	-0.728	0.068
β_3 - Solar radiation$_{sib}$	**-0.291**	**-0.472**	**-0.088**	-0.282	-0.712	0.122
β_4 - Predawn leaf water potential$_{sib}$	-	-	-	-	-	-
β_5 - Midday leaf water potential$_{sib}$	-	-	-	-	-	-
β_6 - Nshoots$_{sib}$	-	-	-	-	-	-
β_7 - CWD : Lifeform.Tree $_{sib}$	-	-	-	**1.317**	**0.768**	**1.919**
β_8 - Lifeform.Tree : Solar radiation$_{sib}$	-	-	-	**0.731**	**0.15**	**1.316**
**Conditional one inflation probability - γ**	**Model G**			**Model H**		
β_0 - Intercept$_{sib}$	**6.599**	**2.35**	**11.17**	**10.653**	**6.093**	**16.877**
β_1 - Lifeform Tree $_{sib}$	-0.339	-5.181	4.398	-	-	-
β_2 - CWD$_{sib}$	**4.544**	**1.691**	**7.894**	**-3.628**	**-6.491**	**-1.112**
β_3 - Solar radiation$_{sib}$	**-2.063**	**-4.071**	**-0.433**	**-4.94**	**-9.275**	**-1.367**
β_4 - Predawn leaf water potential$_{sib}$	-	-	-	-	-	-
β_5 - Midday leaf water potential$_{sib}$	-	-	-	**-1.381**	**-2.719**	**-0.249**
β_6 - Nshoots$_{sib}$	**4.133**	**1.874**	**6.713**	**4.682**	**1.819**	**8.242**
β_7 - CWD : Lifeform.Tree$_{sib}$	-	-	-	-	-	-
β_8 - Lifeform.Tree : Solar radiation$_{sib}$	**3.118**	**1.004**	**5.776**	-	-	-

See notes in the next page

Median is the median estimate for the fixed effects calculated from the posterior distribution. Fixed effects were estimated for both the continuous (the mean [μ] and precision [ν] of the beta distribution) and discrete (the zero-one-inflation [α] and the conditional one-inflation probability [γ]) processes of the ZOIB model. Parameter estimates for μ, α and γ are on the logit scale and for ν on the log (base e) scale. '89CI lower' and '89CI upper' are the lower and upper 89% credible interval limits, respectively. Credible intervals were computed using the highest density interval (HDI) of posterior distributions, which is recommended for non-symmetric (posterior) distributions (Kruschke, 2014). $\beta_{0...8}$ represent the estimated coefficients for μ, ν, α and γ parameters of the ZOIB regression from Equation 2 in Methods S3. The reference level for Lifeform is 'liana' (lianas = 0, trees = 1). b is branch, i is individual and s is species. For each forest, we constructed candidate models by removing model terms that did not contribute to the quality of the models and the best fit model was selected using leave-one-out cross-validation. Covariates that did not contribute to the model are indicated by "-". A coefficient that contains zero within the CIs indicates a negligible association between the covariate and the response variable at the community level (fixed effect; this table) but suggests an important interspecific variation in the response (random effect [slope]; Table S5). Random effects are in Table S5.

Table S5. Summary of the random effects for the models that best-fitted the proportion of leaf cover.

Summary of the random effects for the models that best-fitted the proportion of leaf cover on branches of lianas and trees in the dry (models A C, E and G, PNM) and wet (models B, D, F and H, BPSL) forest.

	**Dry forest - PNM**			**Wet forest - BPSL**		
**Random effects**	σ	**89CI lower**	**89CI upper**	σ	**89CI lower**	**89CI upper**
μ	**Model A**			**Model B**		
S_s	0.483	0.153	0.801	0.318	0.028	0.526
$\tau_s - CWD$	0.454	0.294	0.637	0.226	0.115	0.343
$\delta_s - Srad$	0.276	0.162	0.394	0.355	0.232	0.494
$I_{i(s)}$	0.315	0.006	0.558	0.233	0	0.416
$B_{b(si)}$	0.702	0.536	0.857	0.727	0.63	0.83
ν	**Model C**			**Model D**		
S_s	0.184	0	0.427	0.436	0.09	0.738
$\tau_s - CWD$	0.419	0.081	0.789	0.836	0.486	1.246
$\delta_s - Srad$	0.283	0.062	0.51	0.44	0.099	0.747
$I_{i(s)}$	0.137	0	0.318	0.309	0.001	0.541
$B_{b(si)}$	0.485	0.28	0.708	0.655	0.492	0.839
α	**Model E**			**Model F**		
S_s	0.543	0.051	0.928	0.793	0.431	1.24
$\tau_s - CWD$	0.417	0.203	0.683	0.477	0.166	0.8
$\delta_s - Srad$	0.301	0.063	0.525	0.513	0.209	0.835
$I_{i(s)}$	0.715	0.403	1.061	0.454	0.116	0.802
$B_{b(si)}$	0.574	0.317	0.829	0.293	0.001	0.502
γ	**Model G**			**Model H**		
S_s	3.279	0.008	5.808	3.369	0.019	6.617
$\tau_s - CWD$	4.981	2.059	8.276	2.719	0.687	5.155
$\delta_s - Srad$	0.988	0.001	2.561	5.049	2.081	8.833
$I_{i(s)}$	2.92	0.577	5.707	2.344	0.02	4.914
$B_{b(si)}$	1.338	0	2.8	1.134	0	2.424

σ is the median estimate of the (random effects) standard deviation calculated from the posterior distribution. '89CI lower' and '89CI upper' are the lower and upper 89% credible interval limits, respectively; computed using the highest density interval (HDI) of posterior distributions, which is recommended for non-symmetric (posterior) distributions (Kruschke, 2014). μ is the mean, ν is the precision, α is the zero-one-inflation probability and γ is the conditional one inflation probability of the ZOIB model. S_s is the random intercept for species. τ_s is the species-level random slope of cumulative water deficit (CWD). δ_s is the species-level random slope of solar radiation. $I_{i(s)}$ is the random intercept for individuals nested within species. $B_{b(si)}$ is the random intercept for branch nested within individual and species. Fixed effects are in Table S4.

Methods S1. Estimation of potential evapotranspiration (PET) for the dry Parque Natural Metropolitano (PNM) and the wet Bosque Protector San Lorenzo (BPSL) forest.

We used the Penman formulation (Penman, 1948) implemented in the 'R' package 'Evapotranspiration' (Guo *et al.*, 2019) via the function 'ET.Penman' to estimate daily potential evapotranspiration (PET) for PNM and BPSL. We used the estimated PET to calculate CWD (cumulative water deficit) for each census date and forest site but not for comparisons between sites.

For both forests, we used as input variables hourly values for mean relative humidity (%), solar radiation ($Mj.m^{-2}.day^{-1}$), temperature (Celsius), and wind speed ($m.s^{-1}$). Data was provided by STRI's Physical Monitoring Program and obtained from a permanent weather station at each canopy crane. Data for wind speed for the PNM canopy crane is not available since 2008. Therefore, we used data provided by the Panama Canal Authority from the Albrook Airbase (FAA) weather station, located 2.8km (straight-line) SSW of the PNM crane.

We defined as input constants for each forest the elevation above sea level (30m for PNM and 130m for BPSL), the latitude in radians, and the albedo (Alpha) of the evaporative surface, which represents the portion of incident radiation that is reflected back at the surface. Alpha was set to 0.20 for PNM and 0.15 for BPSL as suggested for deciduous and evergreen broad-leaf forests, respectively (McMahon *et al.*, 2013). For the constants latent heat of vaporization and the Stefan-Boltzmann constant, we used the default values defined in the function 'ET.Penman'.

The following figure shows in the vertical axes the daily PET estimates for PNM (top panel) and BPSL (bottom panel), and in the horizontal axes the date.

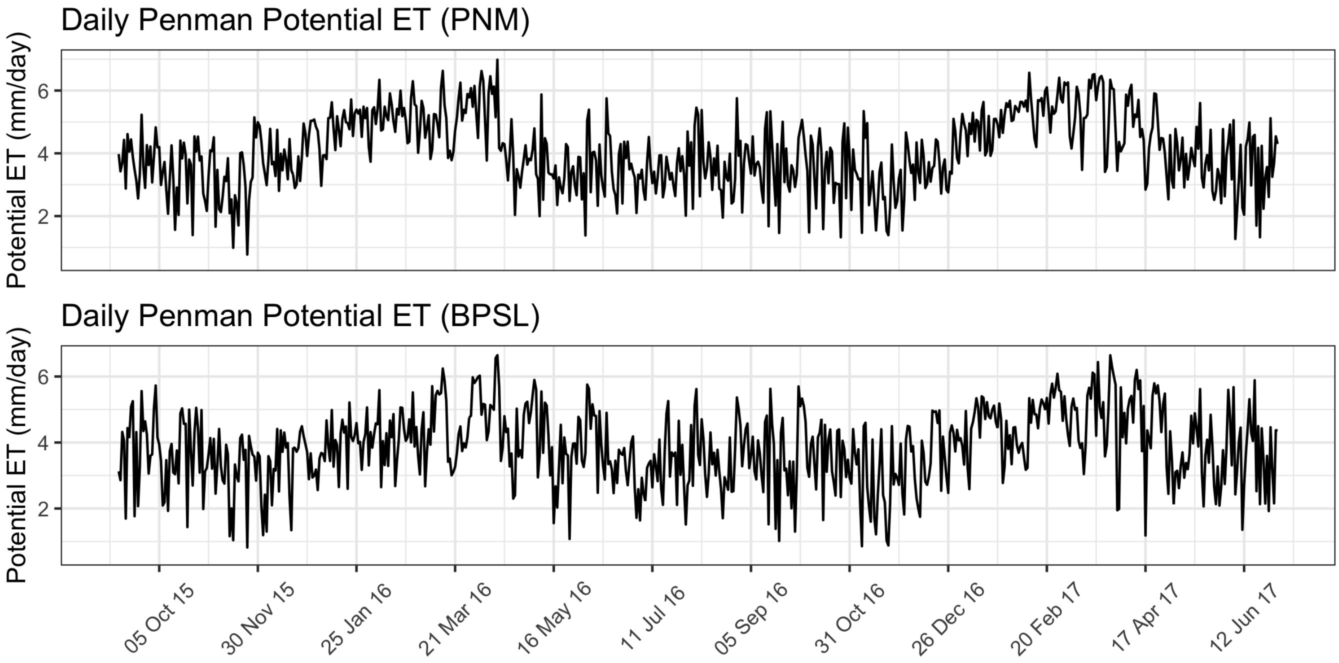

Methods S2. Regression equation of the model for the analysis of predawn and midday leaf water potentials.

We used multilevel models with normally distributed errors to assess the seasonal dynamics of leaf water potentials and the most complex model for each forest had the following form:

Equation 1:

$$\psi_{sic} = \beta_0 + \beta_1 Lifeform_{sic} + \beta_2 CWD_{sic} + \beta_3 Lifeform_{sic} \text{ x } CWD_{sic} + \\ \tau_{i(s)} + \lambda_s + S_s + I_{i(s)} + C_c + \sigma_{sic}$$

ψ_{sic} is the negative of the square root of $|\psi_{pd}|$ or $|\psi_{md}|$ for individual i of species s in census c. A square-root transformation was used to normalize the absolute values of the response variables, predawn (ψ_{pd}) and midday leaf water potential (ψ_{md}). We completed the transformation by multiplying the transformed values by negative one to retain the original direction of the response, with more negative values indicating more negative leaf water potentials. β_0 is the intercept. β_1 is the main effect of the two-level factor lifeform (lianas versus trees). The reference level for lifeform in all models is lianas (lianas = 0, trees = 1). Therefore, negative best-fit values are consistent with hypotheses 1 and 2 (see *Introduction* in the main text). β_2 is the main effect of CWD. β_3 is the interaction between $Lifeform$ and CWD. Positive best-fit values of β_3 indicate ψ_{sic} is more sensitive to CWD_{sic} in trees than in lianas and is also consistent with hypotheses 1 and 2. $\tau_{i(s)}$ and λ_s are the individual- ($i(s)$) and species-level (s) random slopes of CWD, respectively. S_s is the random intercept for species. $I_{i(s)}$ is the random intercept for individuals nested within species. C_c is the random intercept for census. σ_{sic} is the residual standard deviation and is assumed to be independent for different s, i and c. The code to implement the models is available in Zenodo (Medina-Vega *et al.*, 2022)

Methods S3. Regression equation of the model for the analysis of the proportion of leaf cover.

We used Zero/One Inflated Beta Regression (ZOIB) (Ospina & Ferrari, 2008; Liu & Eugenio, 2018) to analyze the seasonal dynamics of the non-normally distributed proportion of leaf cover.

For each forest, the most complex model for each component of the ZOIB regression had the following form:

Equation 2:

$$\begin{aligned} Y_{sib} = \beta_0 + \beta_1 Lifeform_{sib} + \beta_2 CWD_{sib} + \beta_3 Srad_{sib} + \beta_4 \psi_{pd_{sib}} + \beta_5 \psi_{md_{sib}} + \beta_6 sb_{sib} + \\ \beta_7 Lifeform_{sib} \text{ x } CWD_{sib} + \beta_8 Lifeform_{sib} \text{ x } Srad_{sib} + \\ \tau_s + \delta_s + S_s + I_{i(s)} + B_{b(si)} \end{aligned}$$

Y_{sib} is μ (mean), ν (precision), α (zero-one-inflation probability) or γ (conditional one-inflation probability) of branch b of individual i of species s. A logit-link was used for μ, α and γ, and a log-link for ν. β_0 is the intercept. β_1 is the main effect of the two-level factor lifeform (lianas versus trees). β_2 is the main effect of CWD. β_3 is the main effect of solar radiation ($Srad$). β_4 is the main effect of predawn leaf water potential (ψ_{pd}). β_5 is the main effect of midday leaf water potential (ψ_{md}). β_6 is the main effect of the number of axillary shoots (sb). β_7 and β_8 are the estimates of the interaction between $Lifeform$ and CWD and between $Lifeform$ and $Srad$, respectively. τ_s is the random slope of CWD at the species-level. δ_s is the random slope of $Srad$ at the species-level. S_s is the random intercept for species. $I_{i(s)}$ is the random intercept for individuals nested within species. $B_{b(si)}$ is the random intercept for branch nested within individuals and species. The code to implement the models is available in Zenodo (Medina-Vega *et al.*, 2022)

Notes S1. Justification for the inclusion of the group-level random intercept ‘census’ in the models that best-fitted Predawn (ψ_{pd}) and Midday (ψ_{md}) leaf water potentials.

In preliminary analyses, we simulated predawn (ψ_{pd}) and midday (ψ_{md}) leaf water potentials for lianas and trees in the dry (PNM) and wet (BPSL) forest using posterior draws from the full model described in Methods S2 without including the random intercept ‘census’. We observed discrepancies between the simulations and the observed data. In the following figure, the black line in each panel indicates the observed leaf water potentials (y) and each of the 100 gray lines represent each simulation (100 draws, y_{rep}). Note that the absolute value of the observed and simulated leaf water potentials were square-root transformed. We completed the transformation by multiplying the transformed values by negative one to get the direction of the original responses.

Differences between observed and simulated leaf water potentials were particularly strong for midday leaf water potentials in both the dry (panel b) and wet (panel d) forests.

We included the categorical variable ‘census’ as an additional random intercept to absorb variation in the global intercept unexplained by only species and individual identity. By including the census number as an additional random intercept in each model, simulations and observed values had higher similarities than including only species and individual identity (Notes S6); indicating that the global intercept do vary from census to census. This improvement in model-fit was particularly strong for the models that best-fitted midday leaf water potentials for both the dry (PNM) and wet (BPSL) forest.

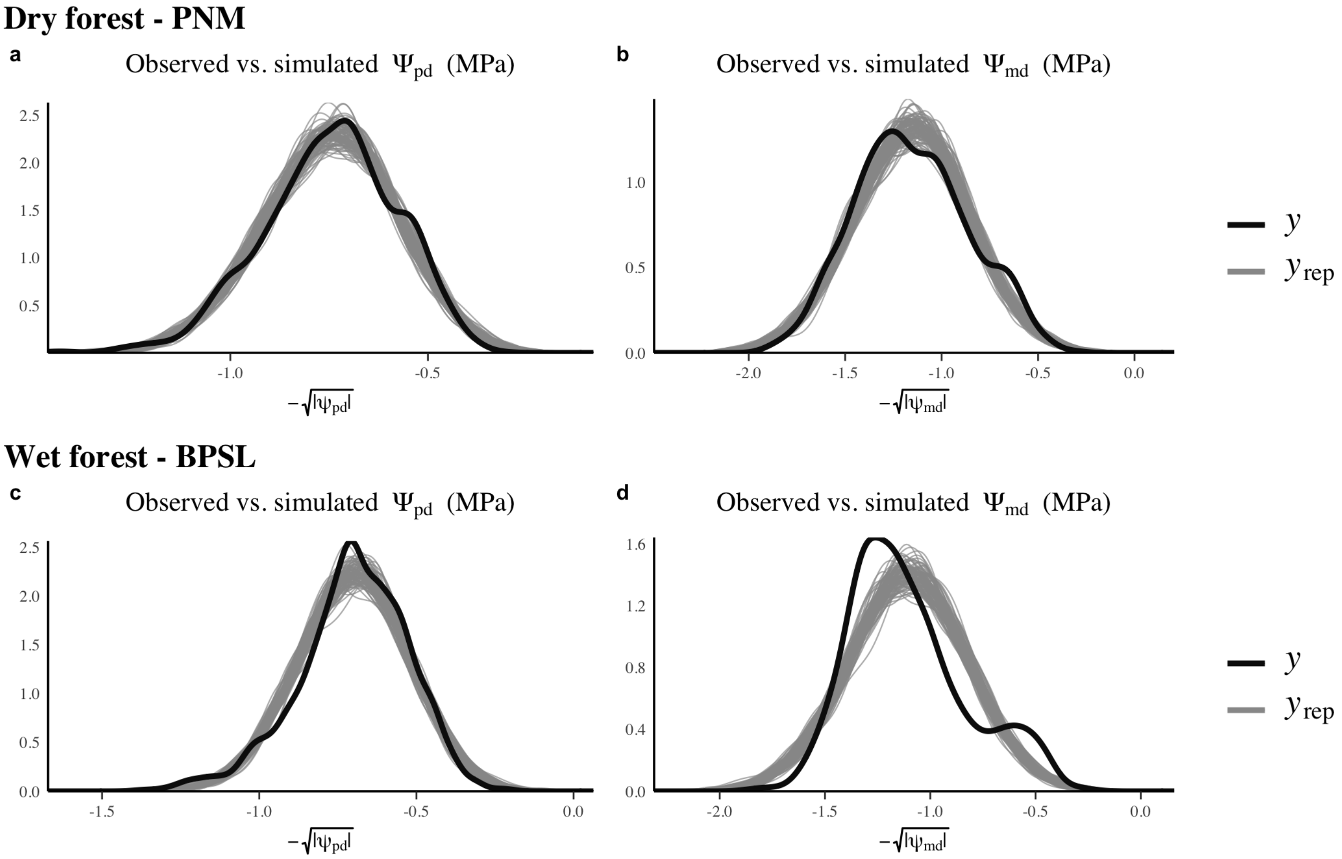

Notes S2. Prior justification and sensitivity analysis for the models that best-fitted Predawn (ψ_{pd}) and Midday (ψ_{md}) leaf water potentials.

For the models that best-fitted predawn (ψ_{pd}) and midday (ψ_{md}) leaf water potentials we used non-informative priors. Non-informative or uninformative priors represents a lack of knowledge about the value of the parameters being estimated. For the community level coefficients (fixed effects), we used a normal distribution with mean (μ) of zero and a standard deviation (σ) of 100, $N(0, 100)$. For the random effects, we used a half student-t prior with three degrees of freedom (shape parameter), zero as the location parameter (μ), and a scale parameter (σ) conditioned on the standard deviation of the response variable. The use of half student-t distribution is supported on the set of all real numbers that are greater or equal to μ $[\mu, \infty)$; appropriate for standard deviations since these are conditioned to be non-negative.

For cases were non-informative priors are used, it is recommended to assess how sensitive are the parameters to prior specifications. We compared the shape of the posterior distributions based on our non-informative prior specification - $N(0, 100)$ - for the fixed effects with posterior distributions of models using flat priors (Uniform), weakly informative priors - $N(0, 10)$ - and more informative priors - $N(0, 1)$.

Dry forest - Parque Natural Metropolitano

Predawn (ψ_{pd}) leaf water potentials

The following figure shows the posterior distribution density for each of the parameters of the best-fitted model for predawn (ψ_{pd}) leaf water potentials in the dry forest coloured conditioned on the prior specification, and indicates that the prior specification has no impact on the posterior distribution.

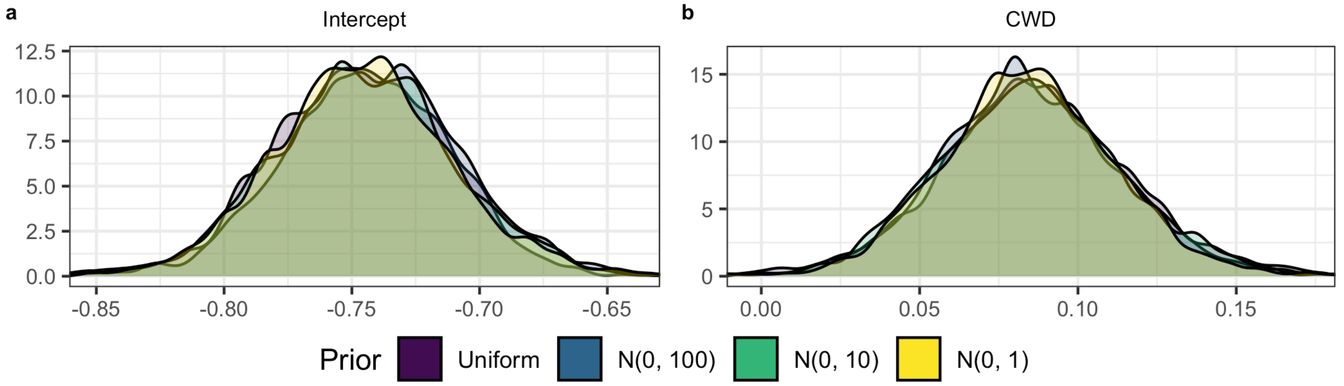

Midday (ψ_{md}) leaf water potentials

For the model that best-fitted midday leaf water potentials in the dry forest, the prior specification has no impact on the posterior distribution.

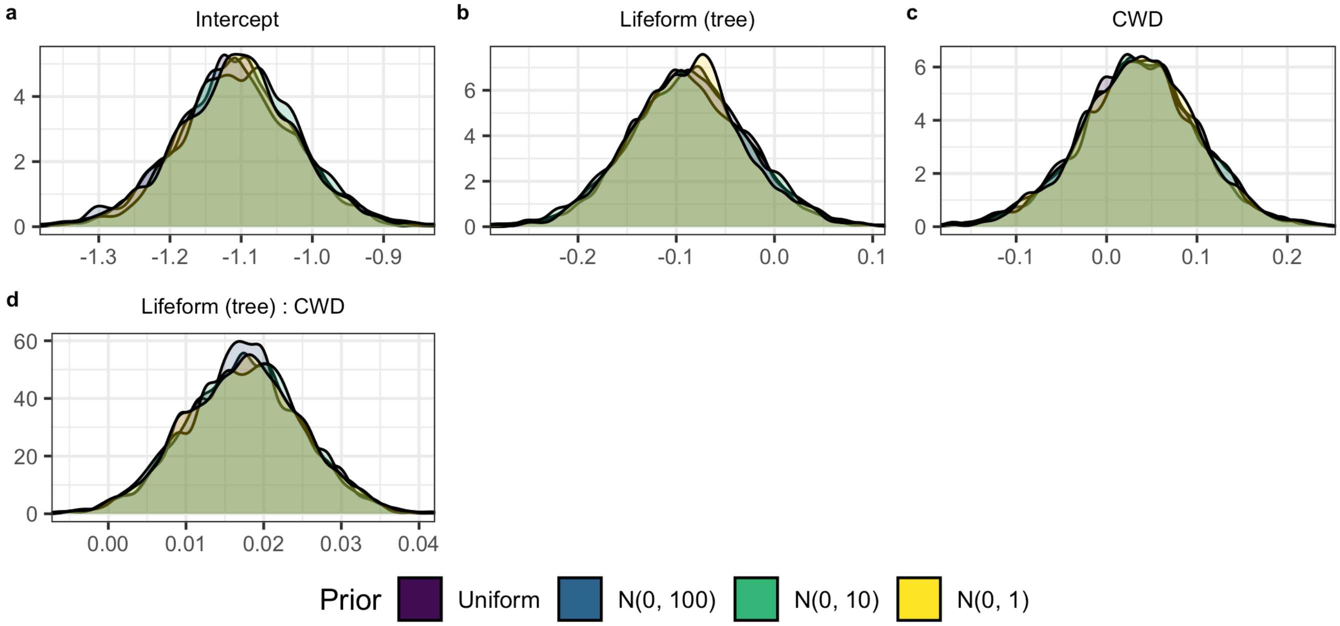

Wet forest - Bosque Protector San Lorenzo

Predawn (ψ_{pd}) leaf water potentials

The following figure shows the posterior distribution density for each of the parameters from the best-fitted model for predawn (ψ_{pd}) leaf water potentials in the wet forest, colored conditioned on the prior specification. The prior specification has no influence on the posterior distribution.

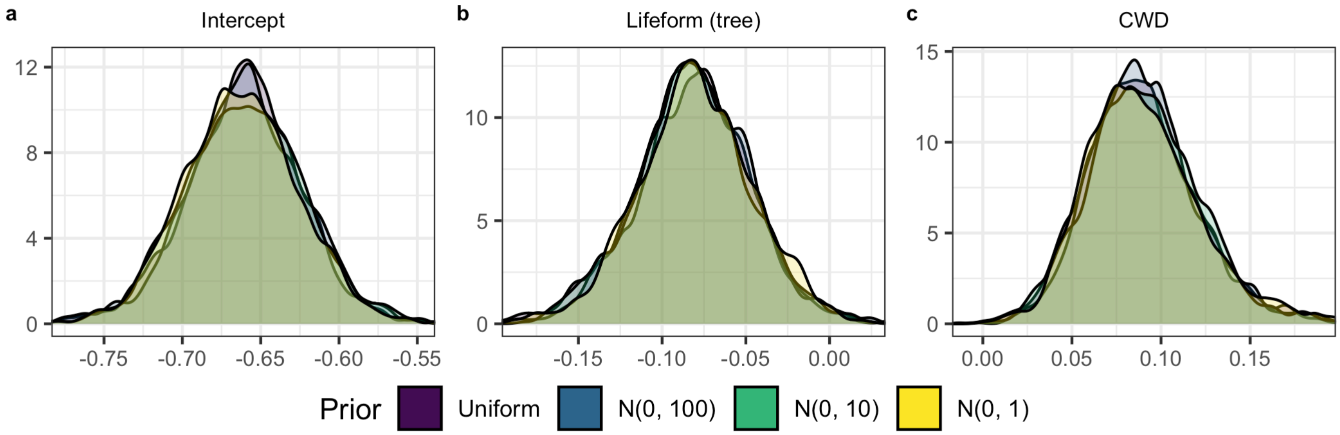

Midday (ψ_{md}) leaf water potentials

The following figure indicates that the posterior distributions of the parameters from the best-fitted model for midday leaf water potentials (ψ_{md}) in the wet forest are no sensitive to prior specifications.

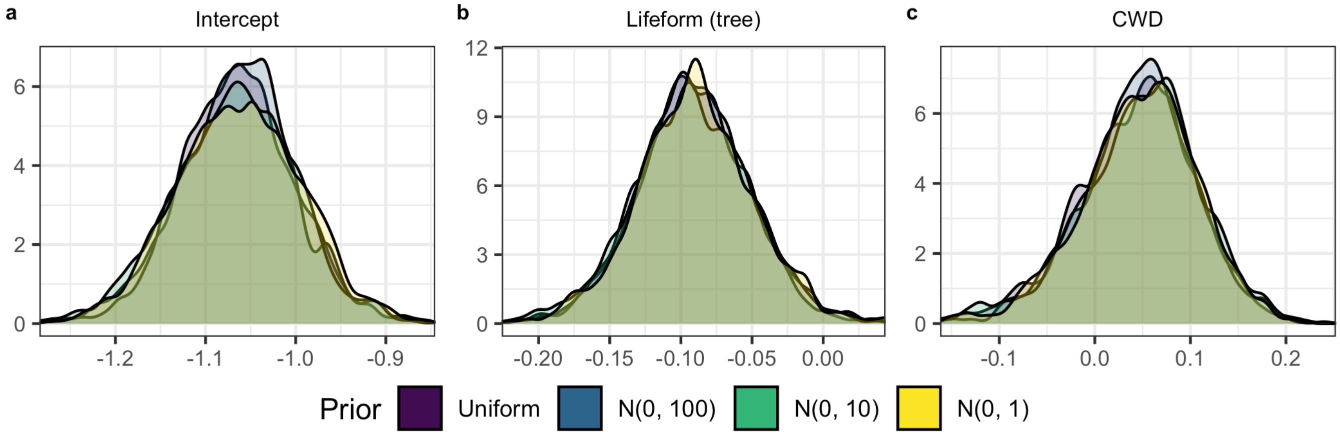

Notes S3. Prior justification and sensitivity analysis for the models that best-fitted the proportion of leaf cover.

We used non-informative priors for the models that best-fitted the proportion of leaf cover and assessed how sensitive are the posterior distributions of the parameters of the best-fitted models to the non-informative prior specifications.

Dry forest - Parque Natural Metropolitano

The following figure (next page) shows the posterior distribution density for each of the fixed effects colored conditioned on the prior specification. Note that all parameters are preceded by the (english) name of the greek letter that represents the continuous and discrete processes of the Zero/One Inflated Beta Regression. mu (μ) is the mean and nu (ν) is the precision of the beta distribution in the ZOIB model. alpha (α) and gamma (γ) are the zero-one-inflation probability and the conditional one-inflation probability of the ZOIB model, respectively. Among all parameters, the Intercept (j), Lifeform (tree) (k), CWD (l), Srad (m), Nshoots (n) and the interaction between Lifeform (tree) and CWD (o) of the zero-one-inflation probability, alpha (α), are the most sensitive to parameter specifications. The normal distribution with mean of 0 and standard deviation of 1 is very constraining and suggest that the use of a normal distribution with mean of 0 and standard deviation of 100 is appropriate since our aim is to obtain answers based on the likelihood function rather than on constrained distributions of unknown parameter values.

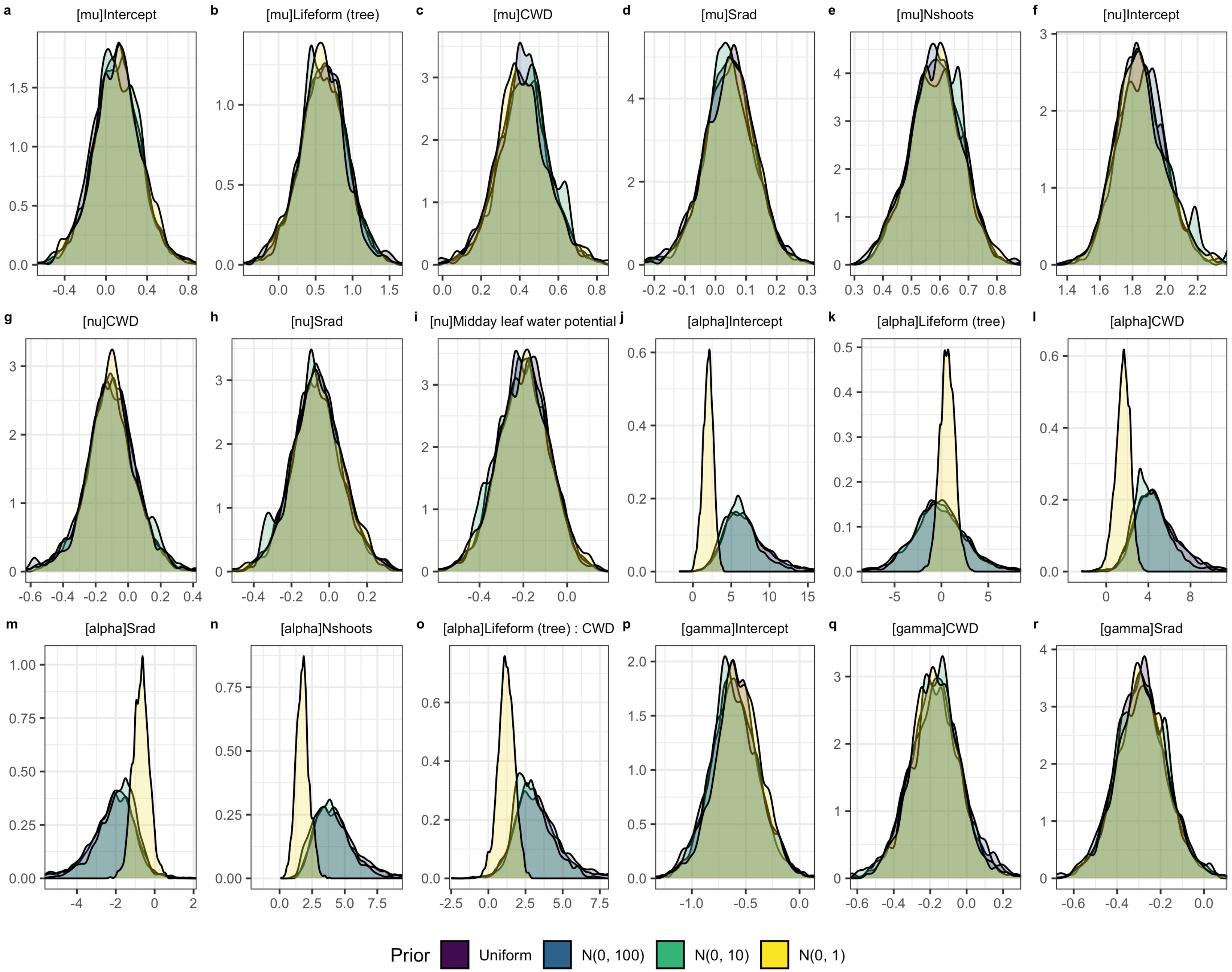
a [mu]Intercept
b [mu]Lifeform (tree)
c [mu]CWD
d [mu]Srad
e [mu]Nshoots
f [nu]Intercept
g [nu]CWD
h [nu]Srad
i [nu]Midday leaf water potential
j [alpha]Intercept
k [alpha]Lifeform (tree)
l [alpha]CWD
m [alpha]Srad
n [alpha]Nshoots
o [alpha]Lifeform (tree) : CWD
p [gamma]Intercept
q [gamma]CWD
r [gamma]Srad
Prior
Uniform
N(0, 100)
N(0, 10)
N(0, 1)

Wet forest - Bosque Protector San Lorenzo

For the wet forest, the Intercept (k), CWD (l), Srad (m), Midday leaf water potentials (n) and Nshoots (o) of the zero-one-inflation probability, alpha (α), are the most sensitive to parameter specifications. The use of the prior normal distribution with $\mu = 0$ and $\sigma = 1$ constraints the shape of posterior distribution and suggests that the use of less informative priors may be more appropriate.

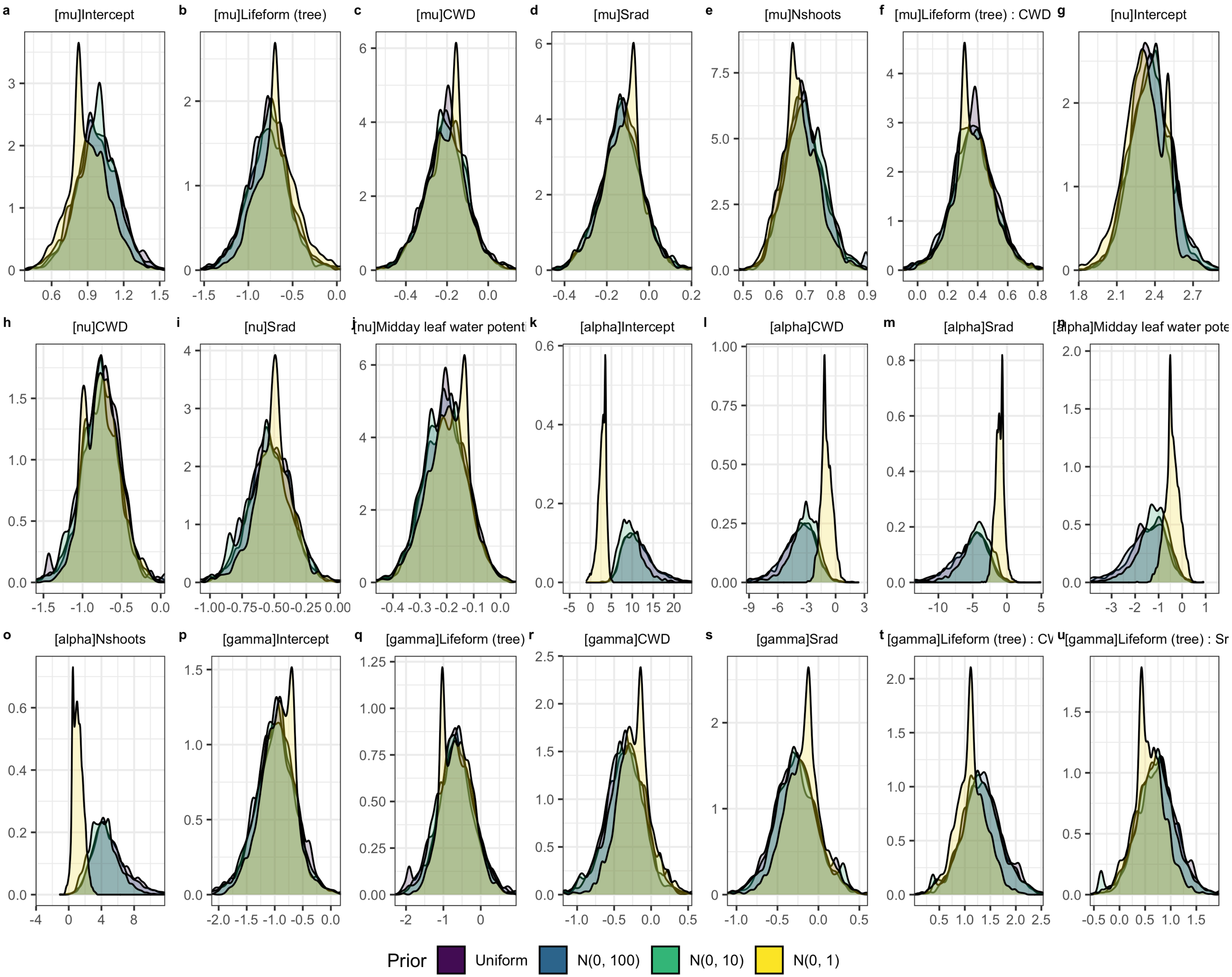
a [mu]Intercept
b [mu]Lifeform (tree)
c [mu]CWD
d [mu]Srad
e [mu]Nshoots
f [mu]Lifeform (tree) : CWD
g [nu]Intercept
h [nu]CWD
i [nu]Srad
j [nu]Midday leaf water potent
k [alpha]Intercept
l [alpha]CWD
m [alpha]Srad
n [alpha]Midday leaf water pote
o [alpha]Nshoots
p [gamma]Intercept
q [gamma]Lifeform (tree)
r [gamma]CWD
s [gamma]Srad
t [gamma]Lifeform (tree) : CV
u [gamma]Lifeform (tree) : Sr
Prior
Uniform
N(0, 100)
N(0, 10)
N(0, 1)

Notes S4. Traceplots for the coefficients of the models that best-fitted Predawn (ψ_{pd}) and Miday (ψ_{md}) leaf water potentials.

Dry forest - Parque Natural Metropolitano

The following figure shows the traceplots for each of the fixed effects of the model that best-fitted predawn (ψ_{pd}) leaf water potentials in the dry forest. The Y axes indicate the values that the parameters took during the runtime of the four chains. The X axes show the 2500 sampling iterations. Warm-up iterations are not included in these plots (2500). The lines with different colors represent each of the four different chains. The figure indicates that the four chains mixed well and suggest satisfactory convergence of the coefficients of the best-fitted model for predawn leaf water potential (ψ_{pd}) in the dry forest.

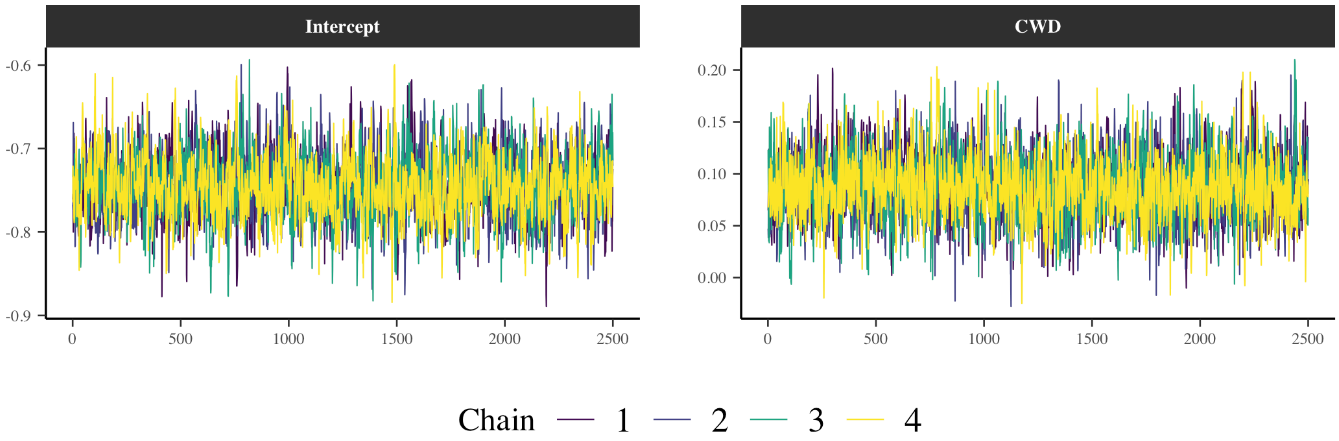

The following figure indicates that the four chains mixed well and suggest satisfactory convergence of the coefficients of the best-fitted model for midday leaf water potential (ψ_{md}) in the dry forest.

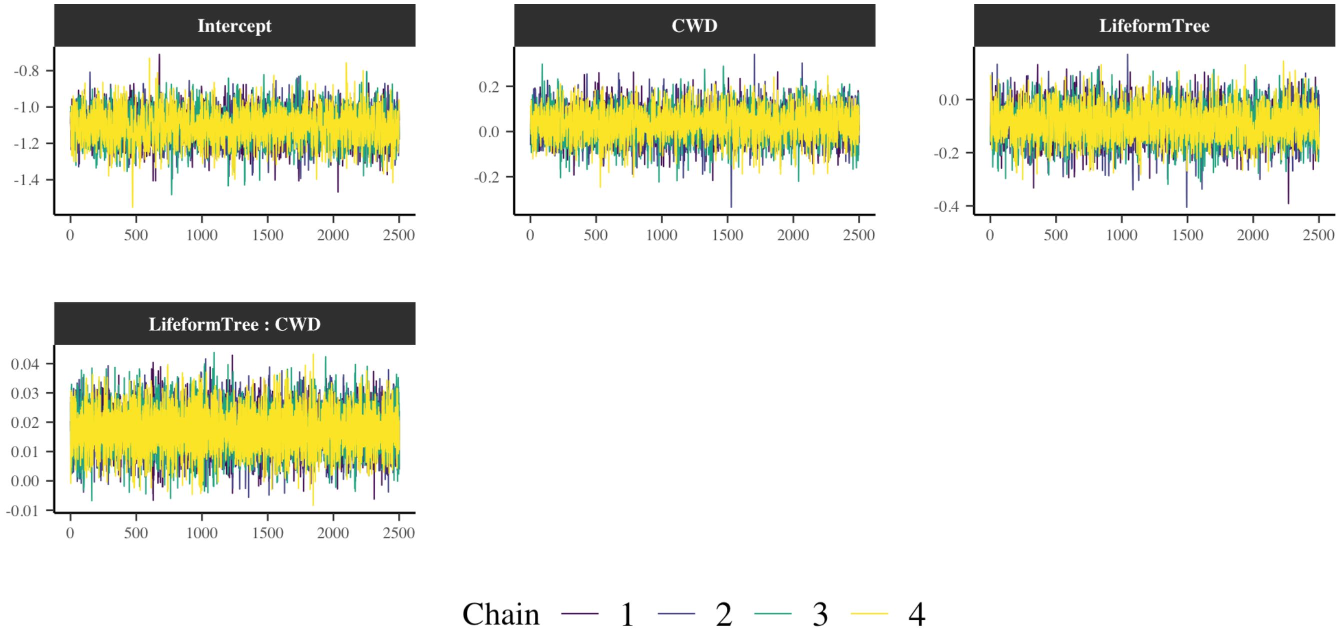

Wet forest - Bosque Protector San Lorenzo

We constructed traceplots for the models that best-fitted predawn leaf water potential (ψ_{md}) in the wet forest. The following figure indicates that the four chains mixed well and suggest satisfactory convergence of the coefficients of the best-fitted model.

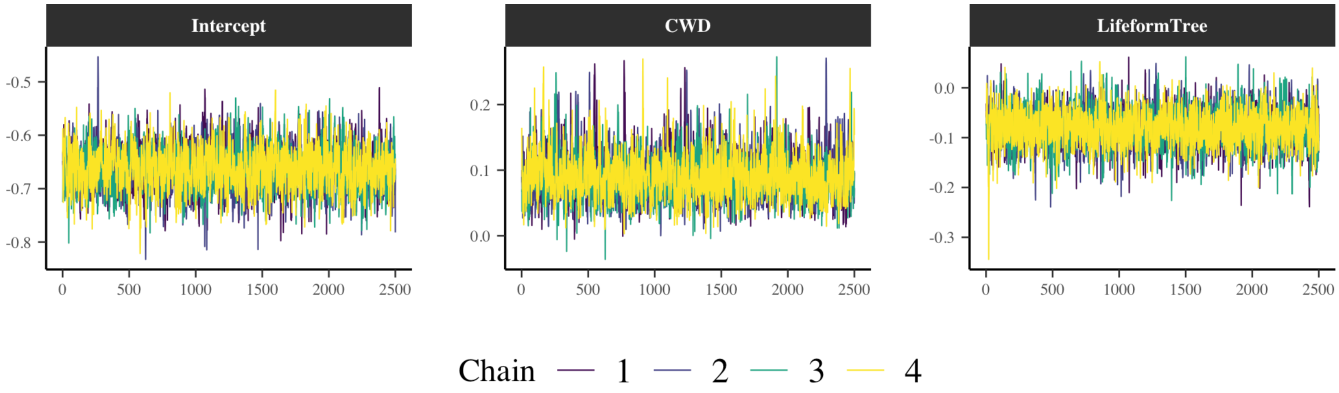

The following figure indicates that the four chains mixed well and suggest satisfactory convergence of the coefficients of the best-fitted model for midday leaf water potential (ψ_{md}) in the wet forest.

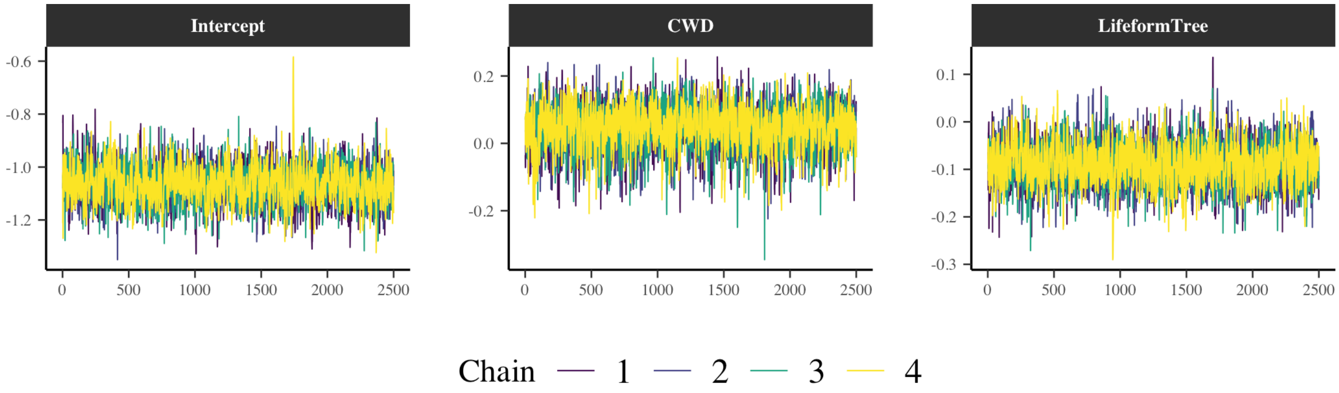

Notes S5. Traceplos for the coefficients of models that best-fitted the proportion of leaf cover.

Dry forest - Parque Natural Metropolitano

The following figure shows the traceplots for each of the community-level coefficients of model that best-fitted the proportion of leaf cover for the dry forest. The Y axes indicate the values that the parameters took during the runtime of the four chains. The X axes show the 2500 sampling iterations. Warm-up iterations are not included in these plots (2500). The lines with different colors represent each of the four different chains. The traceplots indicate that the four chains mixed well and suggest satisfactory convergence of the coefficients.

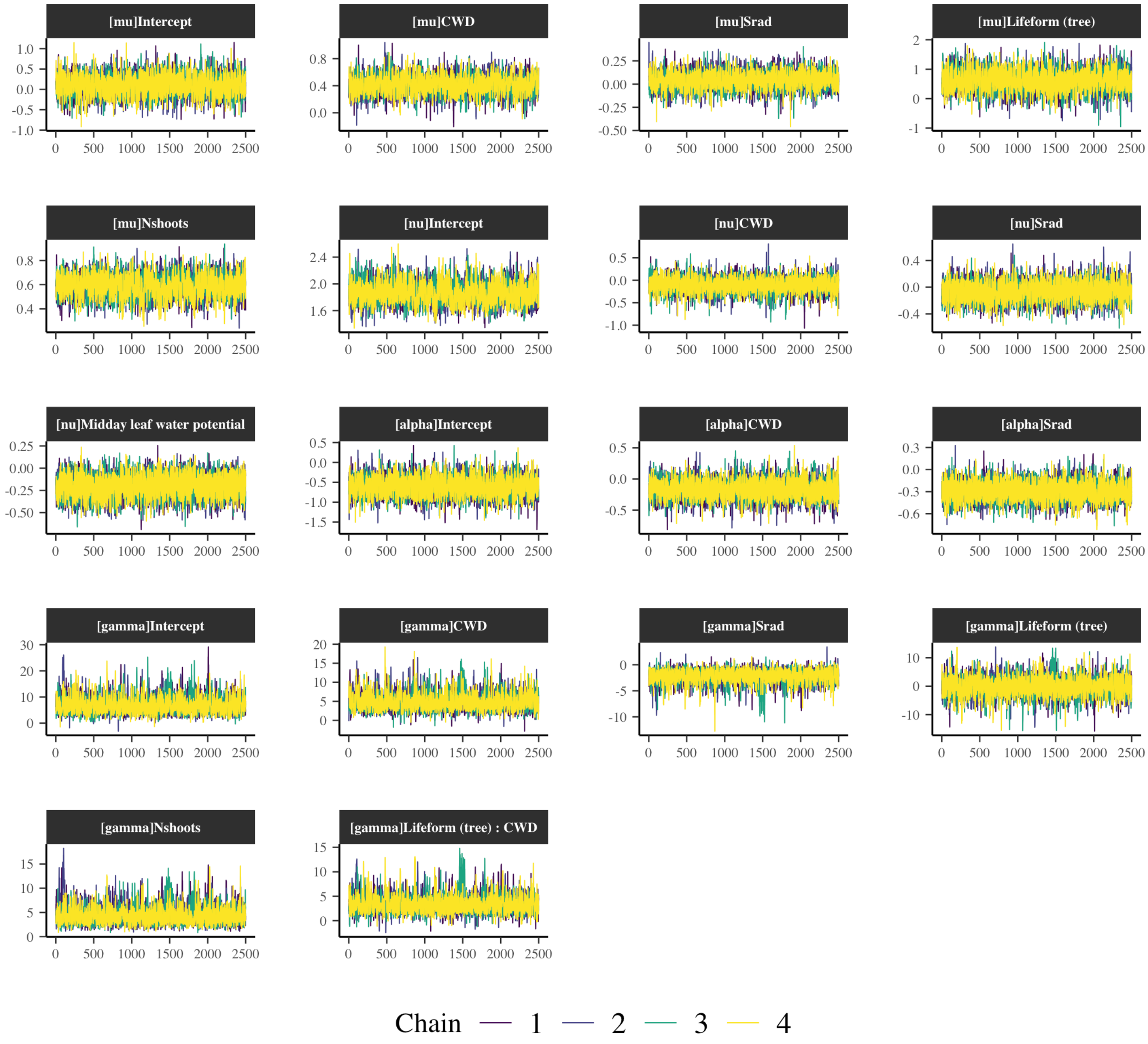

Wet forest - Bosque Protector San Lorenzo

The following traceplots indicate that the four chains mixed well and suggest satisfactory convergence of the coefficients of model that best-fitted the proportion of leaf cover of lianas and trees in the wet forest.

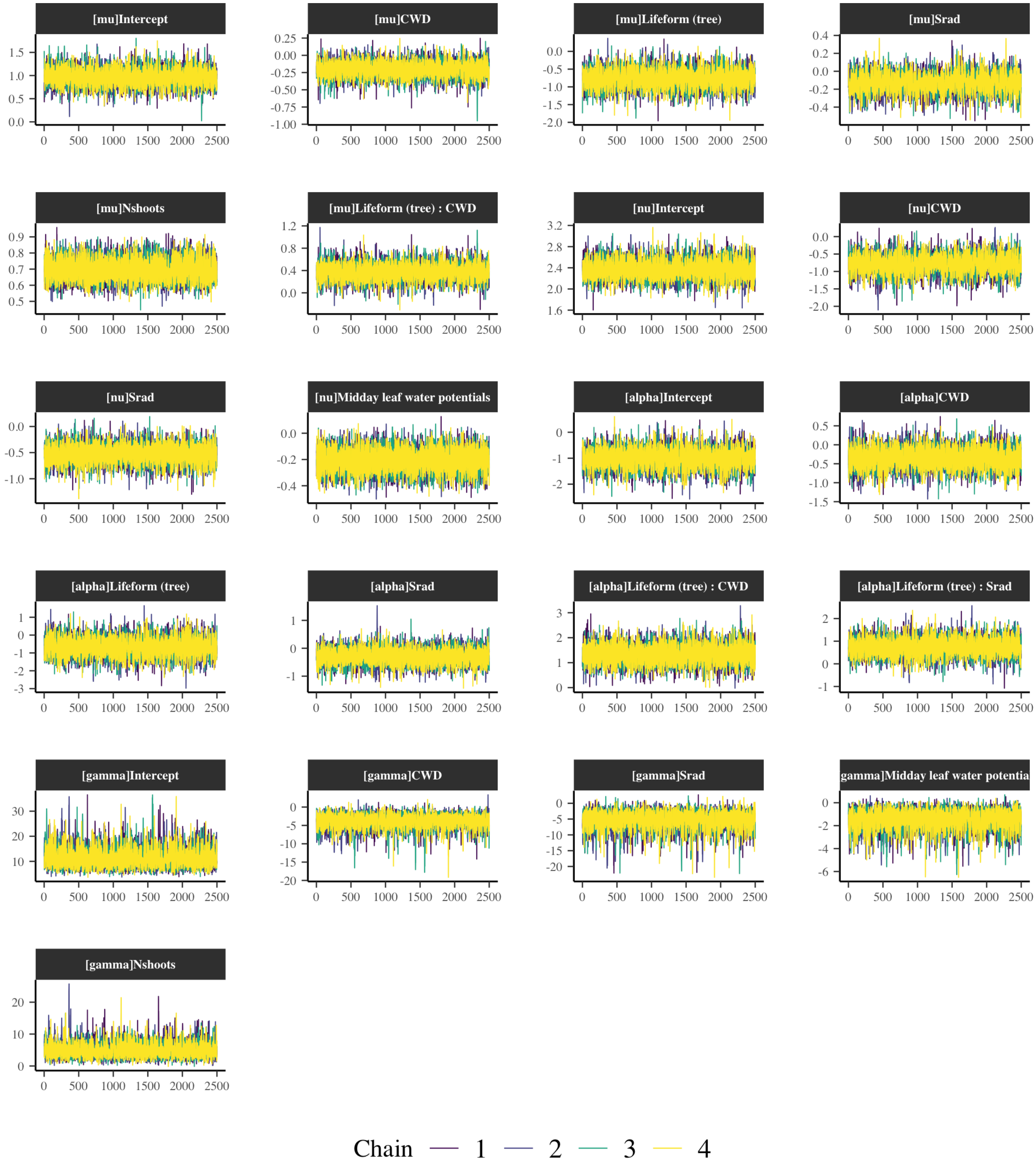

Notes S6. Posterior predictive checks for the models that best-fitted Predawn (ψ_{pd}) and Miday (ψ_{md}) leaf water potentials.

Dry forest - Parque Natural Metropolitano

Using posterior draws from the coefficients of the models that best-fitted predawn (ψ_{pd}) and midday (ψ_{md}) leaf water potentials in the dry forest, we simulated new sets of leaf water potentials and checked if their distribution matched the distribution of the original data. The following figure shows the Kernel density of each dataset for both predawn (panel a) and midday (panel b) leaf water potentials in the dry forest. The black line in each panel indicates the observed leaf water potential (y) and each of the 100 gray lines represent each simulation (100 draws, y_{rep}). Note that the observed and simulated leaf water potentials were square root transformed since this transformation provided the best fit and improved the linear relationship between variables. The simulations (y_{rep}) closely follow the observed (y) predawn (ψ_{pd}, panel a) and midday (ψ_{md}, panel b) leaf water potentials in the dry forest.

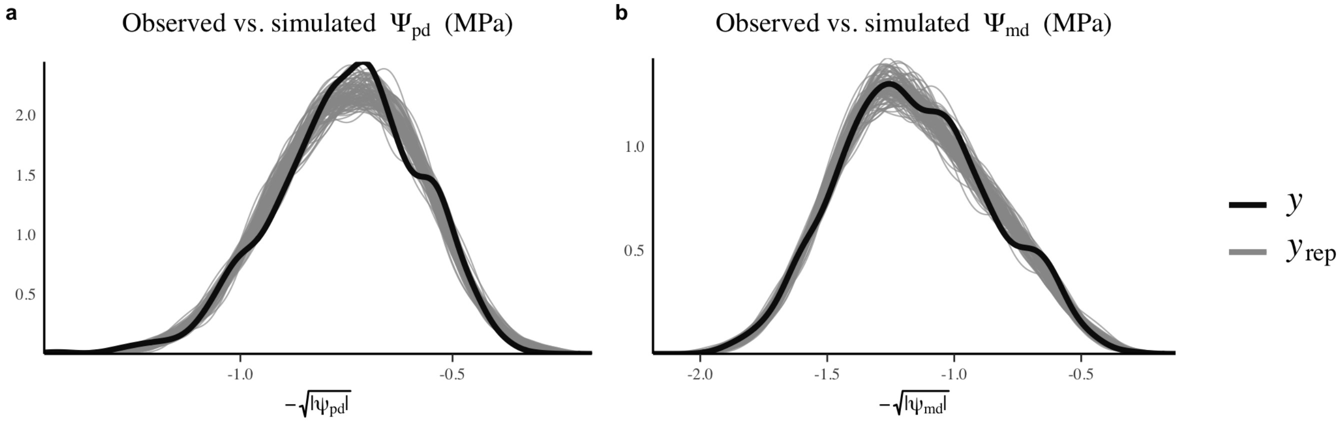

Wet forest - Bosque Protector San Lorenzo

For the wet forest, the simulated (y_{rep}) predawn (ψ_{pd}, panel a) leaf water potentials closely follows the observed (y) values. For midday (ψ_{md}, panel b) leaf water potentials, there is a mismatch between simulations and the observed values, suggesting care in the inference of observed leaf water potential values close to zero.

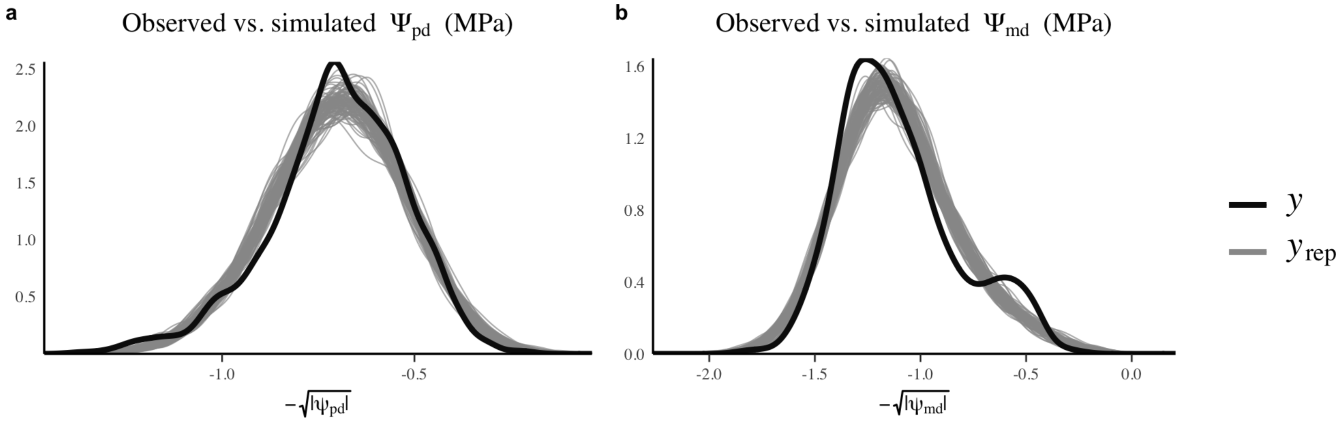

Notes S7. Posterior predictive checks for the models that best-fitted the proportion of leaf cover.

Using posterior draws from the coefficients of the model that best-fitted the proportion of leaf cover, we simulated new sets of data for each forest site and checked if their distribution matched the distribution of the original data. The following figure shows the Kernel density of each dataset for both the dry (panel a) and wet (panel b) forest. The black line in each panel indicates the observed proportion of leaf cover (*y*) and each of the 100 gray lines represent each simulation (100 draws, y_{rep}).

The simulations (y_{rep}) closely follow the observed (*y*) proportion of leaf cover in the dry (panel a) and wet (panel b) forests.

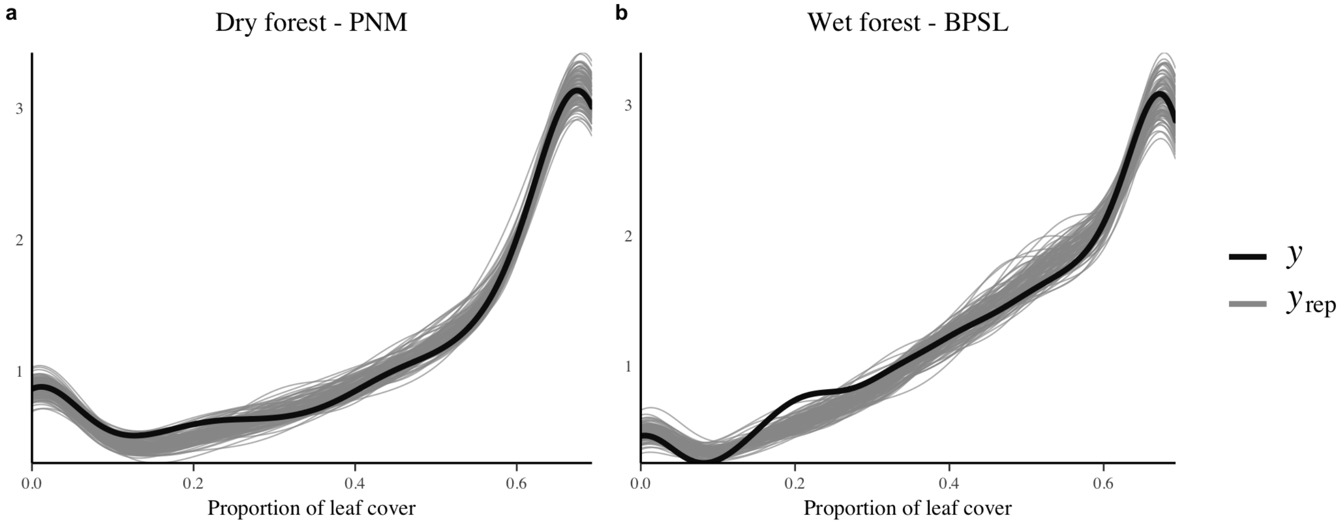

We calculated the proportion of zeros and ones on the original data and simulations. The following figures indicate that the best-fitted models accurately predict the proportion of observed zeros (panels a and b) and ones (panels c and d). The darker vertical lines in each plot shows the proportion of 'observed' zeros (y, panels a and b) and ones (panels c and d). The histograms represent the distribution of the proportion of zeros obtained from 300 draws (y_{rep}) from the posterior predictive distribution.

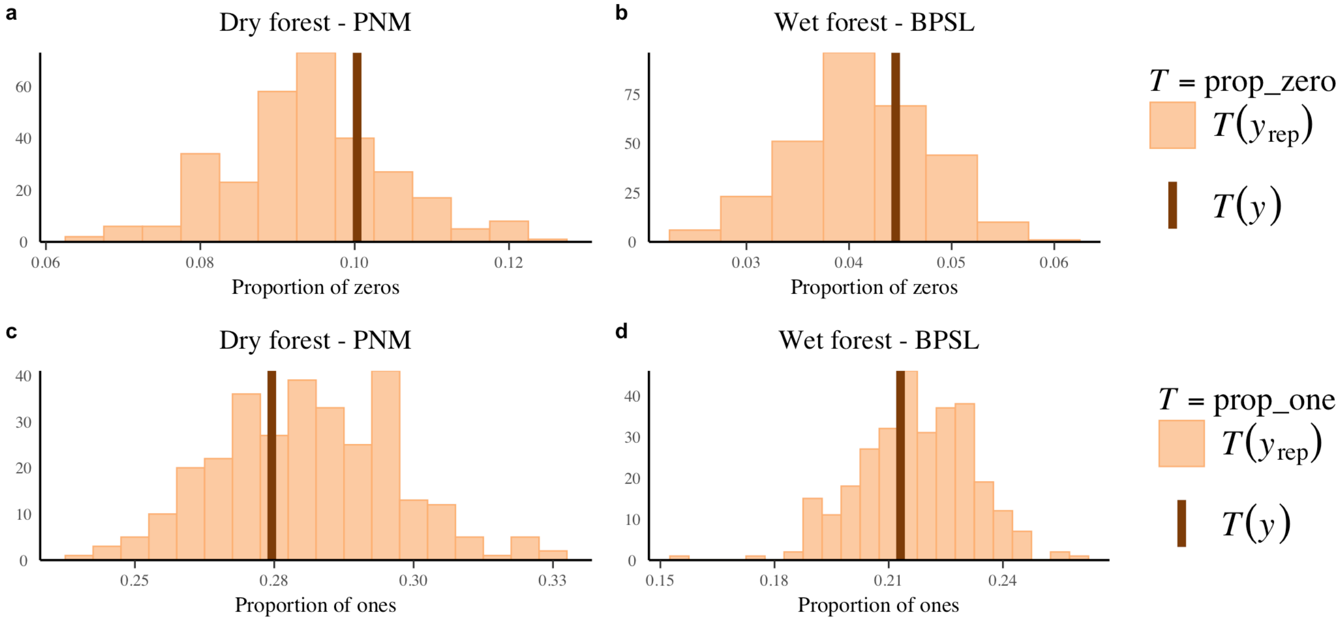

Notes S8. Supplementary results for the seasonal dynamics of leaf cover.

The ZOIB model (refer to the methods section in the main text) analyzes leaf cover proportion data in the closed unit interval [0, 1] and has four parameters, μ and ν for the beta distribution in the (0, 1) interval, or continuous response, and α and γ for the zero and one inflation, or discrete response. The following two subsections describe the four parameters of the ZOIB model fitted for the dry and wet forests.

The seasonal dynamics of leaf cover in the dry, PNM forest

For the continuous response in the (0, 1) interval, proportional leaf cover differed between lianas and trees; however, seasonal changes were statistically indistinguishable for lianas and trees (Fig. 4a [main text]) and were primarily associated with cumulative water deficit (CWD) in both lifeforms. Trees maintained a higher proportion of leaf cover than lianas (Fig. 5a [main text]; Table S4: β_1 in Model A) and lianas and trees both had a higher proportion of leaf cover in wetter periods (Fig. 5b [main text]; Table S4: β_2 in Model A).

For the discrete zero/one response, both lianas and trees were more likely to be fully covered by leaves in wet periods (Table S4: β_2 in Model G) and the few branches that were fully covered by leaves in the dry-season were more likely to be from a tree (Table S4: β_8 in Model G) than from a liana (Table S4: β_3 in Model G). For instance, the brevi-deciduous tree species *Anacardium excelsum* is one of the few species that maintained active leaf production during seasonal drought in the dry forest (Fig. S8). The tree species *Cinnamomum triplinerve* and the liana species *Serjania mexicana* are evergreen and thus maintained a high proportion of leaf cover during seasonal drought. The remeaning seven liana and six tree species are dry-season deciduous and increased leaf cover in wetter periods (Fig. S8).

The seasonal dynamics of cover in the wet, BPSL forest

For the continuous response in the (0, 1) interval, proportional leaf cover and its seasonal changes differed between lianas and trees in the wet forest (Fig. 4b [main text]). Trees had lower proportional leaf cover than lianas (Fig. 5d [main text]; Table S4: β_1 in Model B) and proportional leaf cover increased in wetter periods for trees (Table S4: β_7 in Model B) and in drier periods for lianas (Fig. 5e [main text]; Table S4: β_2 in Model B).

For the discrete zero/one response, trees were more likely to display either a fully covered branch or an empty branch in wetter periods (Table S4: β_7 in Model F) and periods with high light availability (Table S4: β_8 in Model F) while lianas maintained a more consistent branch cover during the whole study period (Table S4: β_2 and β_3 in Model F; Fig. 5e, f [main text]). However, at increasingly high levels of light availability (Table S4: β_3 in Model H), as well as in very wet periods (Table S4: β_2 in Model H), both lianas and trees were less likely to display a fully covered branch.

Fig. S1. Frequency distribution for the proportion of leaf cover.

The following histograms show the frequency distribution of the proportiong of leaf cover on branches of lianas and trees in the dry (PNM, left panel) and wet (BSPL, right panel) forest.

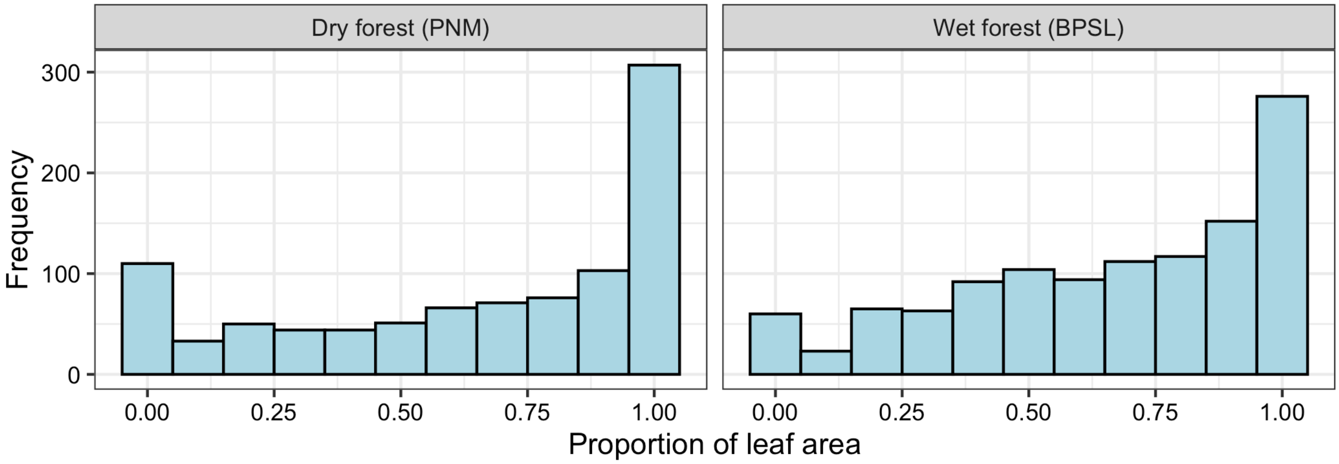

Fig. S2. Conditional effects for the models that best-fitted predawn (ψ_{pd}) and midday (ψ_{md}) leaf water potentials in the dry (PNM) forest.

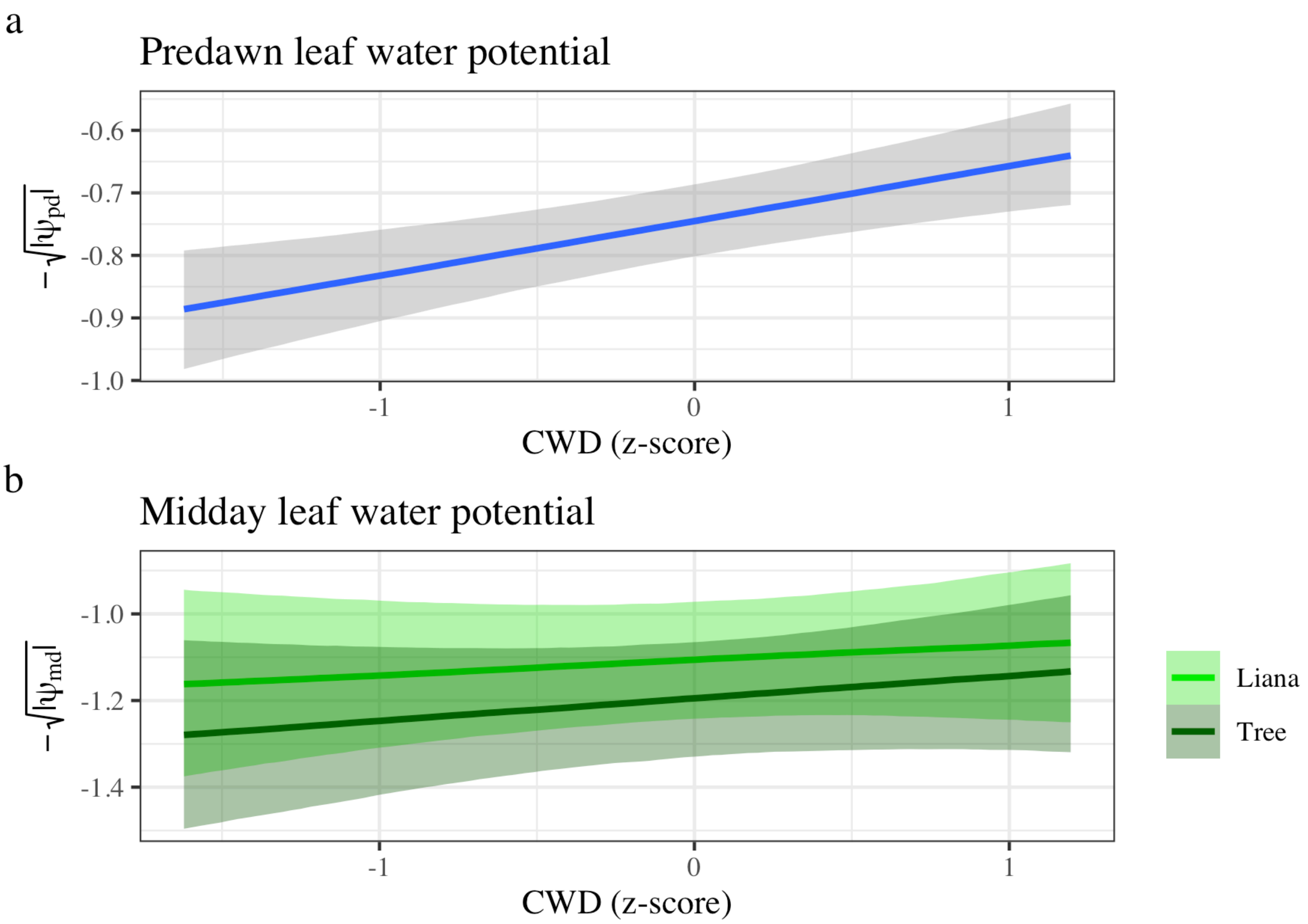

Conditional effects of cumulative water deficit (CWD) on predawn and midday leaf water potential in the dry forest. The Y axes show the median predawn (panel a) and midday (panel b) leaf water potentials. The X axes show the z-score of cumulative water deficit at the time of census (CWD). Blue line in panel a is the predicted median predawn leaf water potential for both lianas and trees. For panel b, light green is the predicted median midday leaf water potential for lianas and dark green is for trees. We used a square root transformation to normalize the absolute value of the observed leaf water potentials. We completed the transformation by multiplying the transformed values by negative one to retain the original direction of the response, with more negative values indicating more negative leaf water potentials. Shadows around the median lines indicate the 89% credible intervals

Fig. S3. Conditional effects for the models that best-fitted predawn (ψ_{pd}) and midday (ψ_{md}) leaf water potentials in the wet (BPSL) forest.

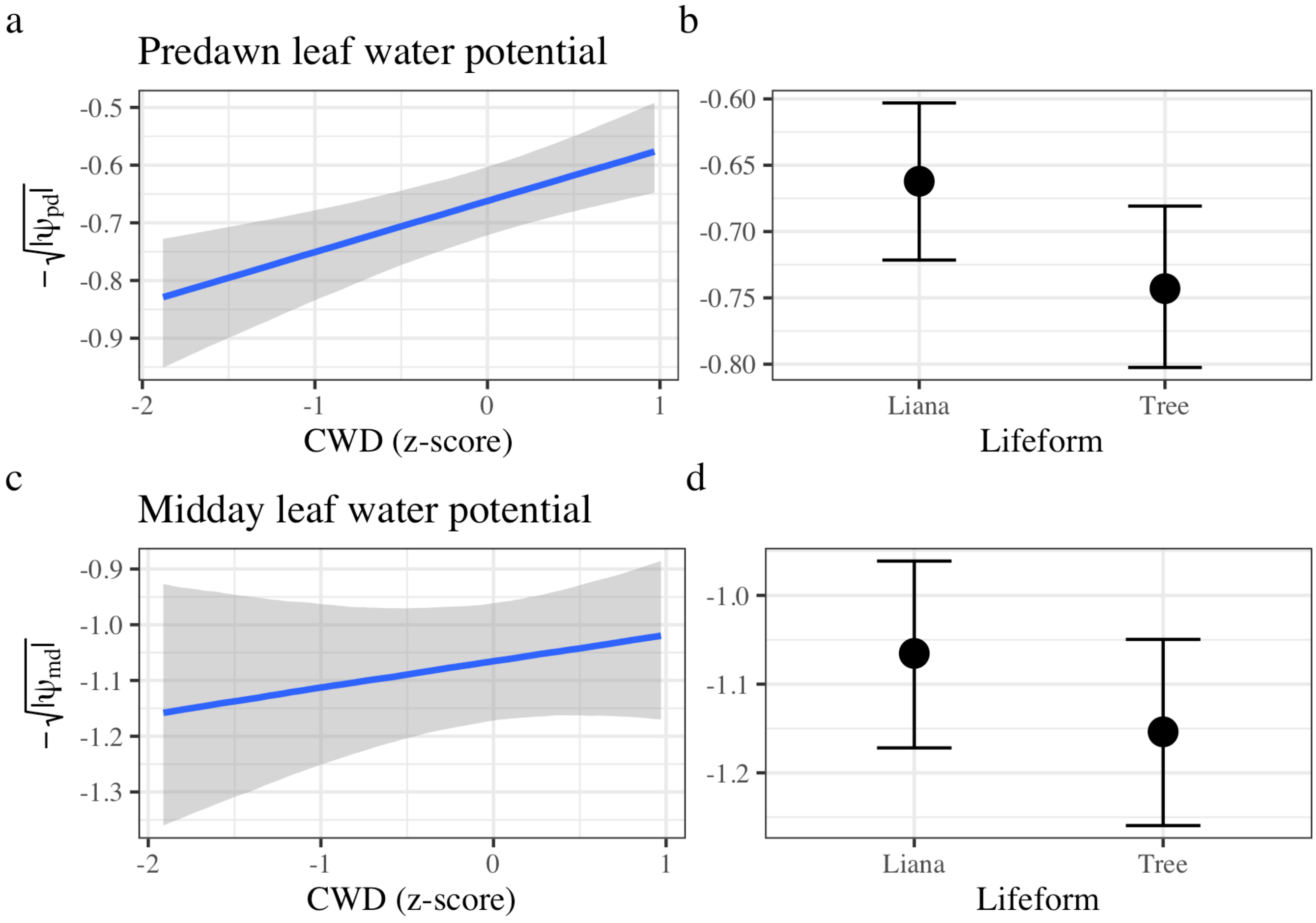

Conditional effects of cumulative water deficit (CWD) and Lifeform on predawn and midday leaf water potential in the wet forest. The Y axes show the predawn (panels a and b) and midday (panels c and d) leaf water potentials. The X axes in panels a and c show the z-score of cumulative water deficit at the time of census (CWD). Te X axes in panels b and d indicate lianas and trees. The blue line in panels a and c indicate the predicted median predawn and midday leaf water potentials for both lianas and trees, respectively. The dots in panels b and d indicate the predicted median predawn and midday leaf water potentials for both lianas (left) and trees (right), respectively. We used a square root transformation to normalize the absolute value of the observed leaf water potentials. We completed the transformation by multiplying the transformed values by negative one to retain the original direction of the response, with more negative values indicating more negative leaf water potentials. Shadows around the median lines in panels a and c and error bars in panels b and d indicate the 89% credible intervals.

Fig. S4. Species-level predictions for predawn leaf water potentials in the dry (PNM) forest.

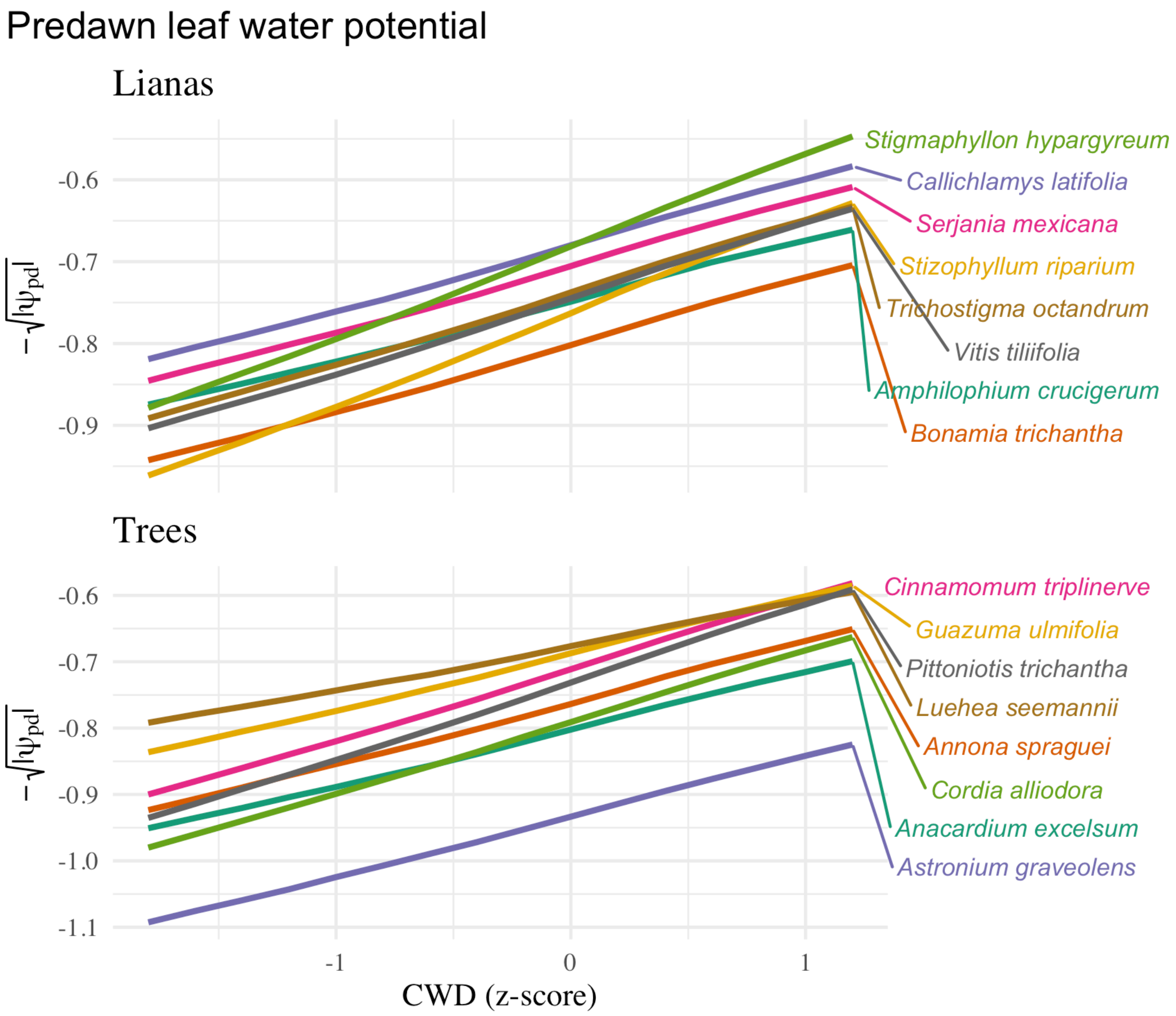

Species-level predictions for predawn leaf water potentials in the dry (PNM) forest to changes in cumulative water deficit (CWD). For each panel, the coloured lines are the predicted median leaf water potentials for each species. Species names correspond to each line. We used a square root transformation to normalize the absolute value of the observed leaf water potentials. We completed the transformation by multiplying the transformed values by negative one to retain the original direction of the response, with more negative values indicating more negative leaf water potentials.

Fig. S5. Species-level predictions for midday leaf water potentials in the dry (PNM) forest.

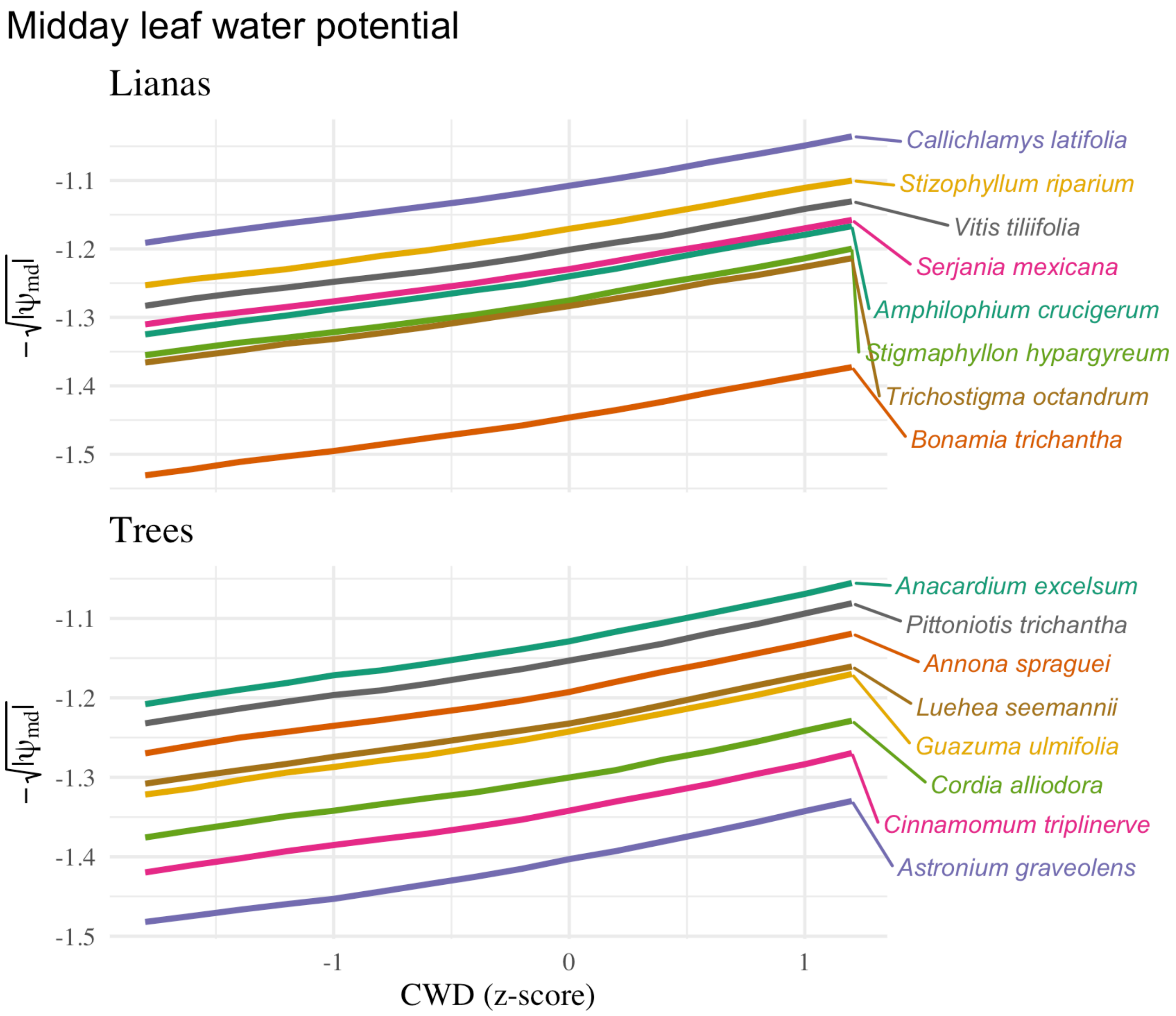

Species-level predictions for midday leaf water potentials in the dry (PNM) forest to changes in cumulative water deficit (CWD). For each panel, the coloured lines are the predicted median leaf water potentials for each species. Species names correspond to each line. We used a square root transformation to normalize the absolute value of the observed leaf water potentials. We completed the transformation by multiplying the transformed values by negative one to retain the original direction of the response, with more negative values indicating more negative leaf water potentials.

Fig. S6. Species-level predictions for predawn leaf water potentials in the wet (BPSL) forest.

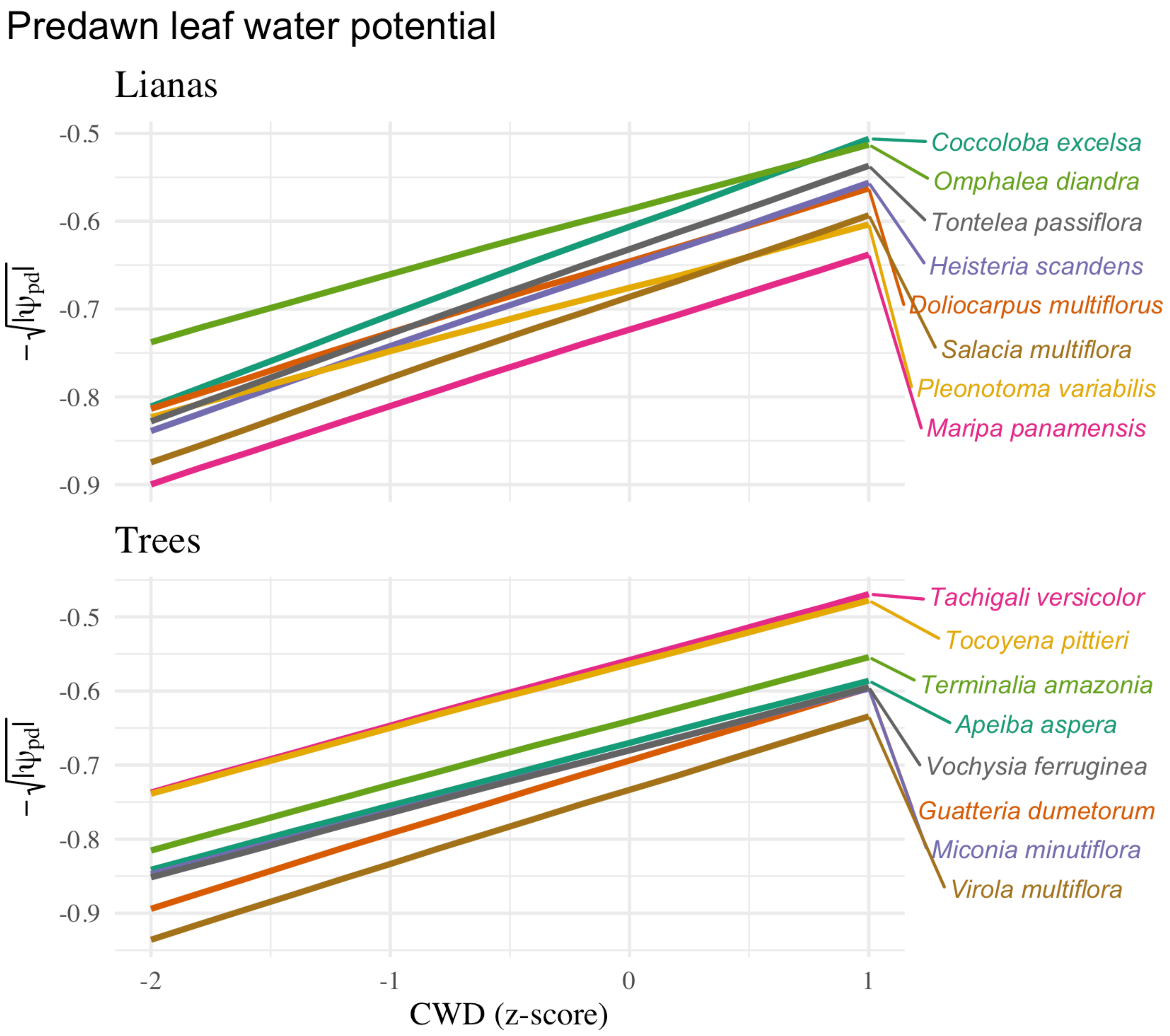

Species-level predictions for predawn leaf water potentials in the wet (BPSL) forest to changes in cumulative water deficit (CWD). For each panel, the coloured lines are the predicted median leaf water potentials for each species. Species names correspond to each line. We used a square root transformation to normalize the absolute value of the observed leaf water potentials. We completed the transformation by multiplying the transformed values by negative one to retain the original direction of the response, with more negative values indicating more negative leaf water potentials.

Fig. S7. Species-level predictions for midday leaf water potentials in the wet (BPSL) forest.

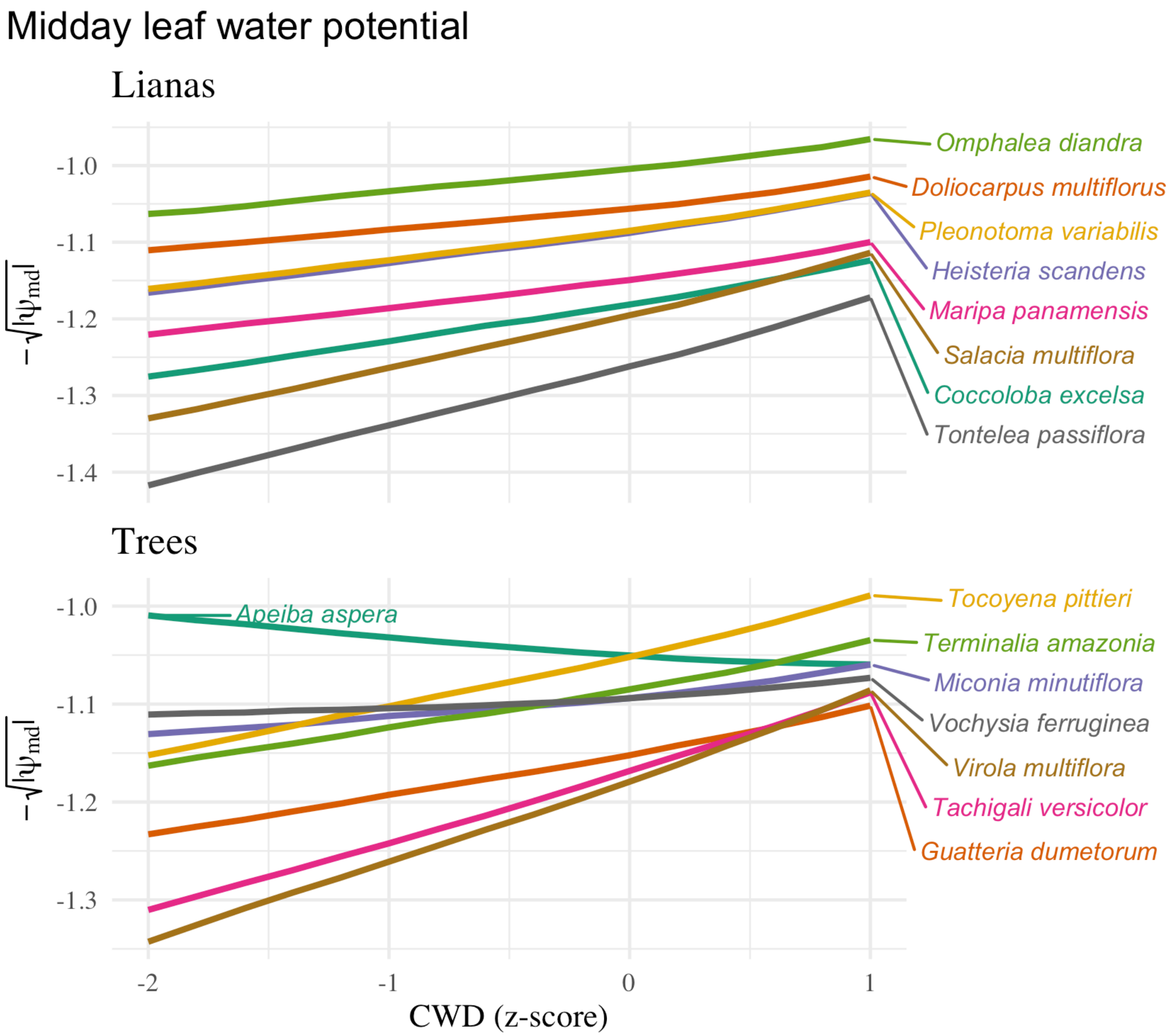

Species-level predictions for midday leaf water potentials in the wet (BPSL) forest to changes in cumulative water deficit (CWD). For each panel, the coloured lines are the predicted median leaf water potentials for each species. Species names correspond to each line. We used a square root transformation to normalize the absolute value of the observed leaf water potentials. We completed the transformation by multiplying the transformed values by negative one to retain the original direction of the response, with more negative values indicating more negative leaf water potentials.

Fig. S8. Species-level predictions for the proportion of leaf cover to changes in cumulative water deficit for the dry (PNM) forest.

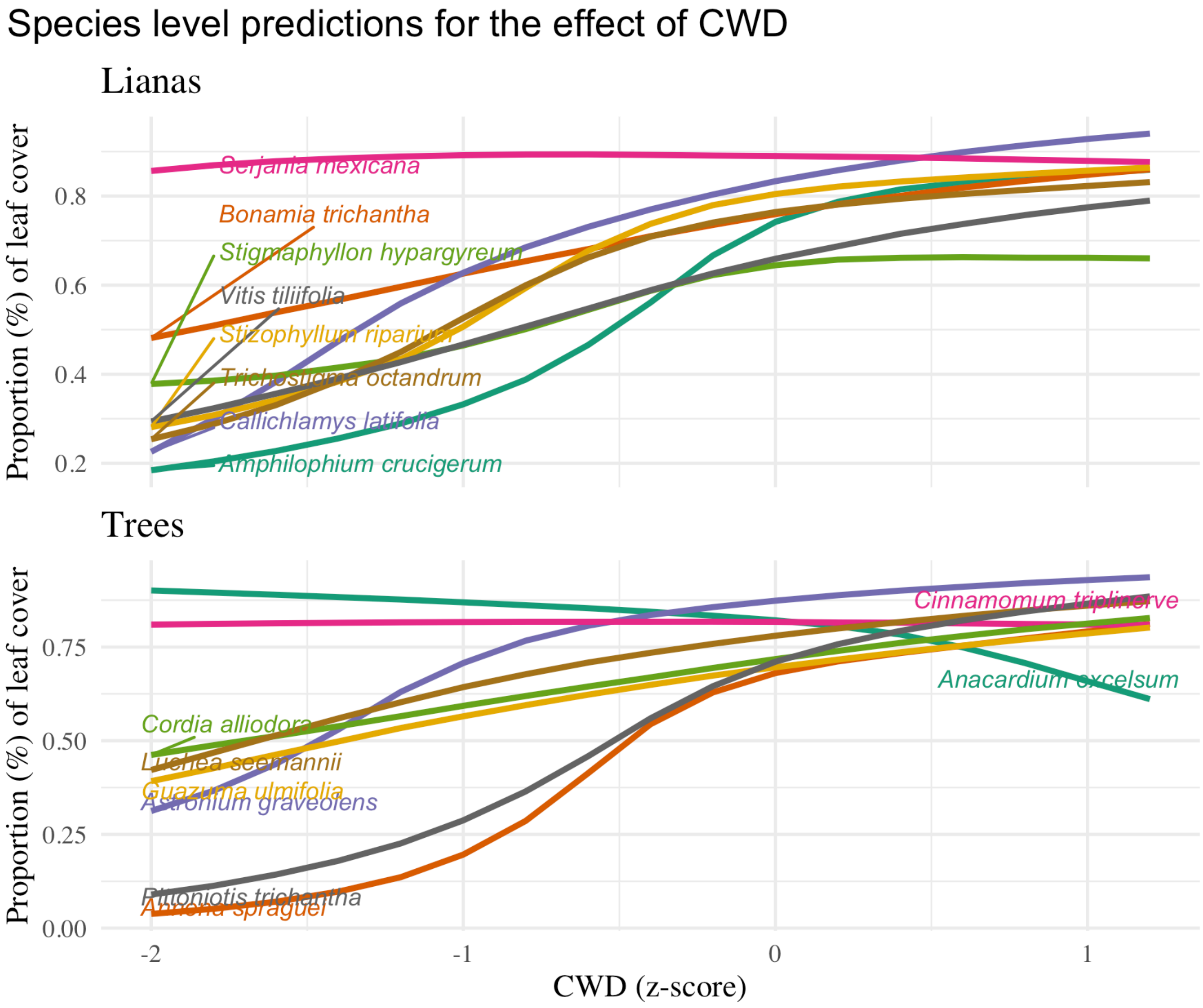

Species-level predictions for the proportion of leaf cover in branches of lianas and trees in the dry (PNM) forest to changes in cumulative water deficit (CWD). The coloured lines are the predicted median proportion of leaf cover for each species. Species names correspond to each line.

Fig. S9. Species-level predictions for the proportion of leaf cover to changes in cumulative water deficit for the wet (BPSL) forest.

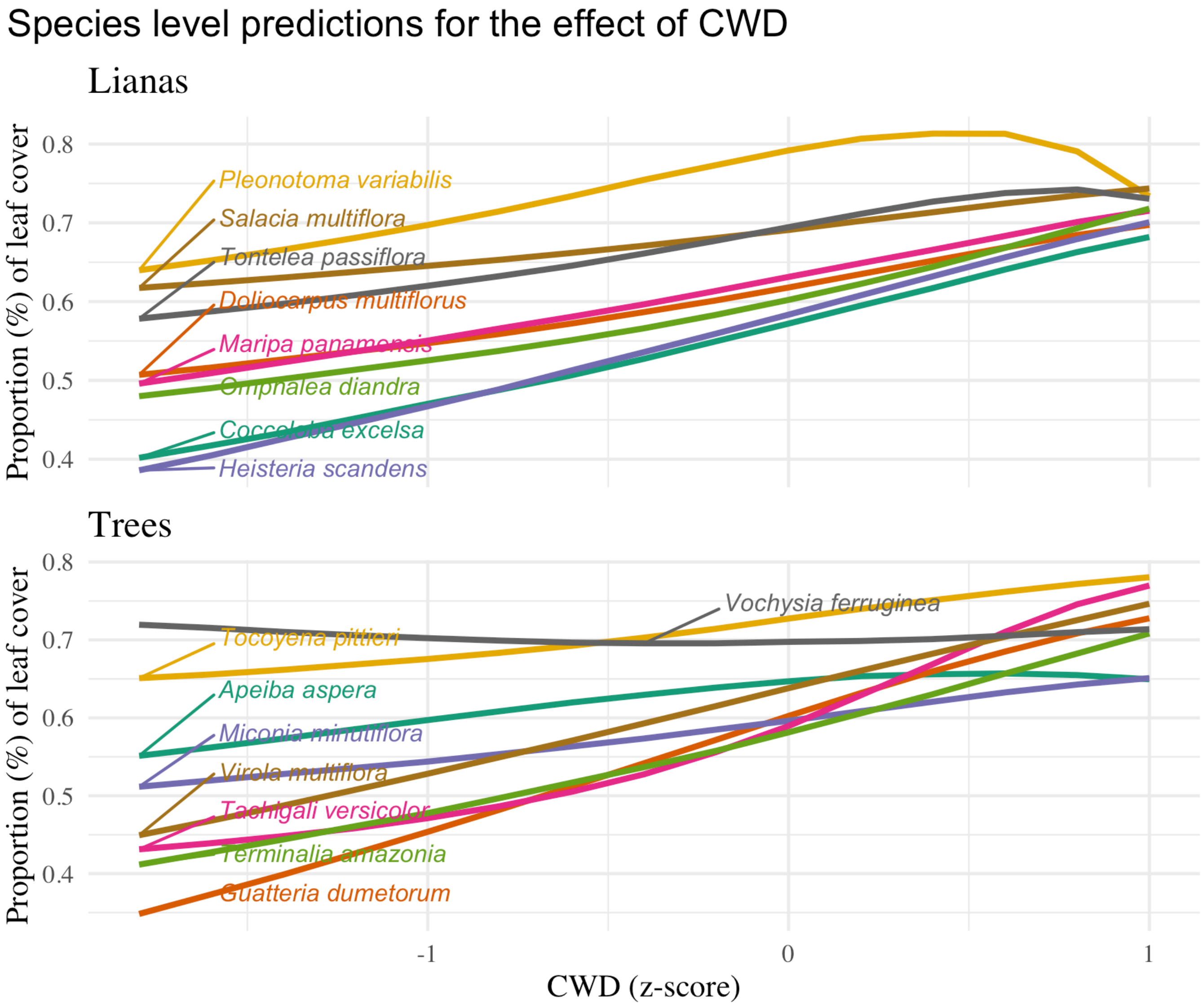

Species-level predictions for the proportion of leaf cover in branches of lianas and trees in the wet (BPSL) forest to changes in cumulative water deficit (CWD). The coloured lines are the predicted median proportion of leaf cover for each species. Species names correspond to each line.

Fig. S10. Species-level predictions for the proportion of leaf cover to changes in solar radiation for the dry (PNM) forest.

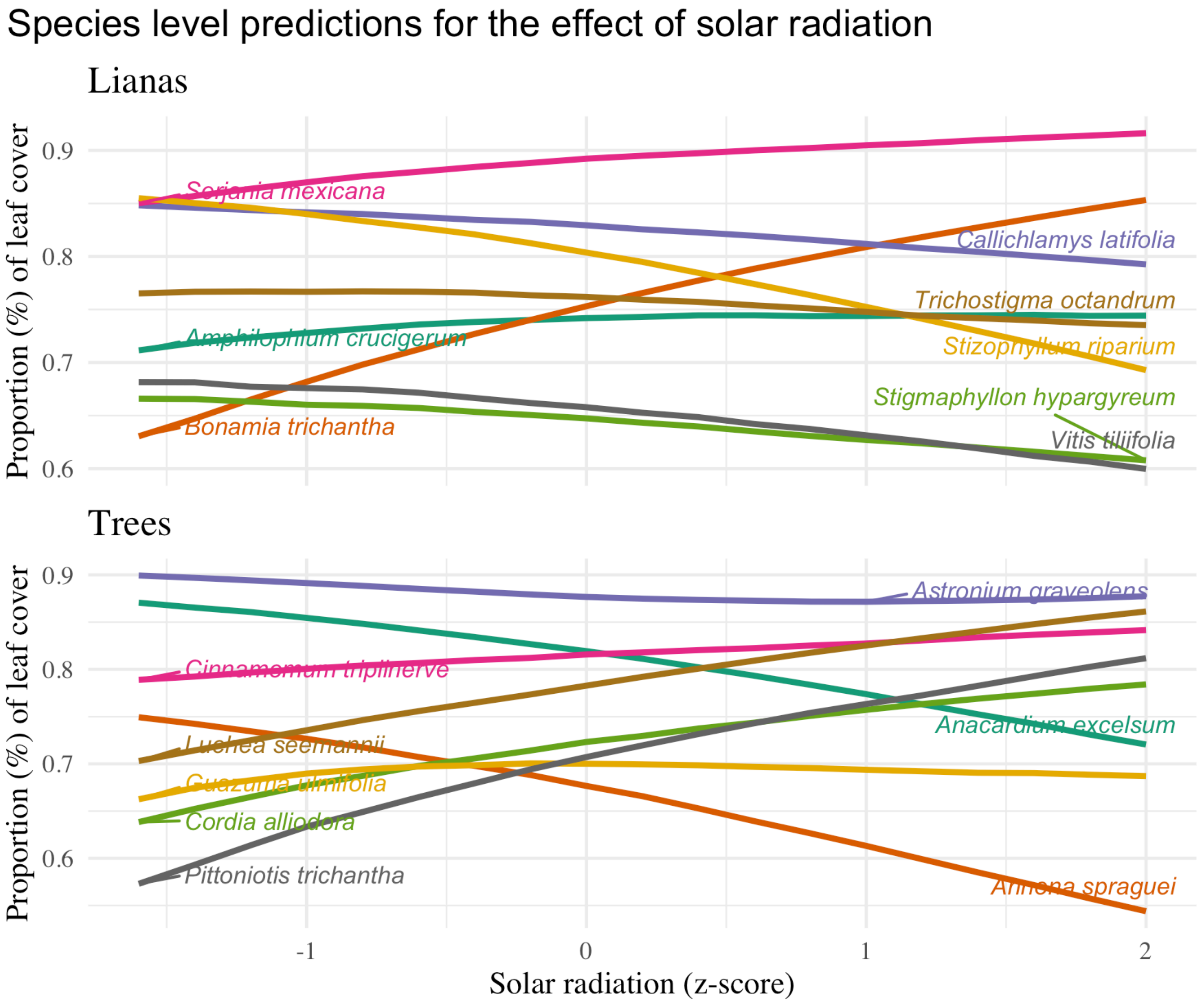

Species-level predictions for the proportion of leaf cover in branches of lianas and trees in the dry (PNM) forest to changes in solar radiation (Srad). The coloured lines are the predicted median proportion of leaf cover for each species. Species names correspond to each line.

Fig. S11. Species-level predictions for the proportion of leaf cover to changes in solar radiation for the wet (BPSL) forest.

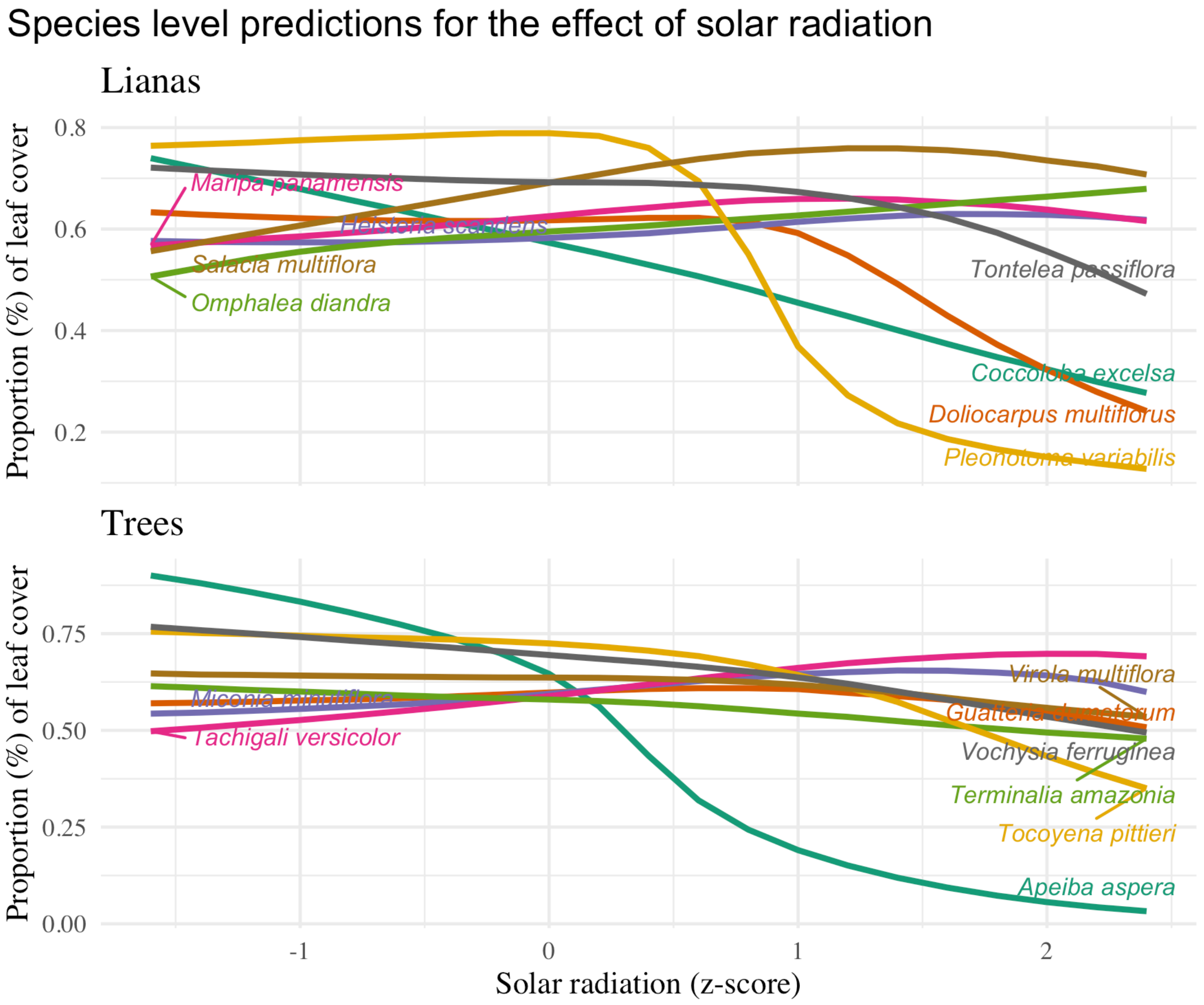

Species-level predictions for the proportion of leaf cover in branches of lianas and trees in the wet (BPSL) forest to changes in solar radiation (Srad). The coloured lines are the predicted median proportion of leaf cover for each species. Species names correspond to each line.

References

Guo D, Westra S, Peterson T. **2019**. *Evapotranspiration: Modelling actual, potential and reference crop evapotranspiration*. R package version 1.14. [WWW document] URL https://CRAN.R-project.org/package=Evapotranspiration. [accessed 11 September 2019].

Kruschke J. **2014**. *Doing bayesian data analysis: A tutorial with r, JAGS, and stan*. Amsterdam, the Netherlands: Academic Press.

Liu F, Eugenio EC. **2018**. A review and comparison of bayesian and likelihood-based inferences in beta regression and zero-or-one-inflated beta regression. *Statistical Methods in Medical Research* **27**: 1024–1044.

McMahon TA, Peel MC, Lowe L, Srikanthan R, McVicar TR. **2013**. Estimating actual, potential, reference crop and pan evaporation using standard meteorological data: A pragmatic synthesis. *Hydrology and Earth System Sciences* **17**: 1331–1363.

Medina-Vega JA, Wright SJ, Bongers F, Schnitzer SA, Sterck FJ. **2022**. *Data from: Vegetative phenologies of lianas and trees in two neotropical forests with contrasting rainfall regimes*. [WWW document] URL https://doi.org/10.5281/zenodo.6403252. [accessed 24 March 2022].

Ospina R, Ferrari SLP. **2008**. Inflated beta distributions. *Statistical Papers* **51**: 111.

Penman HL. **1948**. Natural evaporation from open water, bare soil and grass. *Proceedings of the Royal Society of London. Series A, Mathematical and physical science* **193**: 120–145.
